# Cross-level regional and socioeconomic inequalities in step counts: a descriptive epidemiological investigation of 1.5 million Japanese smartphone users

**DOI:** 10.1016/j.lanwpc.2026.101901

**Published:** 2026-06-19

**Authors:** Kazuaki Arai, Masamitsu Kamada, Takahiro Yoshida, Ikuho Yamada, Ding Ding, Kimihiro Hino

**Affiliations:** a Department of Health Education and Health Sociology, School of Public Health, Graduate School of Medicine, The University of Tokyo, Bunkyo-Ku, Tokyo, Japan; b Center for Spatial Information Science, The University of Tokyo, Kashiwa-shi, Chiba, Japan; c Interfaculty Initiative in Information Science, The University of Tokyo, Bunkyo-Ku, Tokyo, Japan; d Sydney School of Public Health, Faculty of Medicine and Health, The University of Sydney, Sydney, NSW, Australia; e The Charles Perkins Centre, The University of Sydney, Sydney, NSW, Australia; f Department of Urban Engineering, Graduate School of Engineering, The University of Tokyo, Bunkyo-Ku, Tokyo, Japan

**Keywords:** Physical activity, Step counts, Inequalities, mHealth, Walkability, Built environment, Surveillance

## Abstract

**Background:**

Although regional inequalities in physical activity are recognised as a global concern, nationwide evidence from device-based measurements is limited. Given the multilevel nature of inequality, we quantified step count disparities across municipalities and examined cross-level interactions between individual-level socioeconomic status (SES) and walkability.

**Methods:**

We conducted municipality- and individual-level analyses using step count data from 1,542,536 smartphone users aged 20–64 years. We calculated municipality-level mean daily steps and Gini coefficients, and examined associations between mean daily steps, municipal walkability, and Gini coefficients. Linear mixed-effects models quantified cross-level interactions between individual SES (education, employment, and equivalised household income) and walkability (modelled as a continuous variable) on individual-level mean daily steps. Cross-level patterns were visualised across quintile-based walkability strata.

**Findings:**

Municipality-level mean daily steps varied substantially across the 951 municipalities, with a maximum difference of 3724 steps/day (ranging from 4026 to 7750 steps/day, nearly a 2-fold disparity). Municipalities with higher mean daily steps had greater walkability (r = 0.76) and more equal step-count distributions (Gini coefficient; r = −0.79, stronger than simulated mathematical correlations). Significant cross-level interactions on individual-level mean daily steps were evident, with patterns differing across SES domains: step-count differences across walkability strata were prominent for education and income (623–762 steps/day), whereas employment status showed substantial differences between employed and non-employed individuals, reaching 826–1514 steps/day in highly walkable areas.

**Interpretation:**

Substantial inequalities exist across and within municipalities, and individual-level disparities vary based on SES and walkability. Nationwide surveillance and cross-sectoral collaborations are needed to identify populations vulnerable to inactivity and implement targeted strategies.

**Funding:**

Japan Society for the Promotion of Science and Japan Science and Technology Agency.

**Research in context**

**Evidence before this study**

Efforts to reduce the global burden of physical inactivity are hindered by limited population-level evidence on how individual and environmental conditions jointly shape activity patterns. In particular, large-scale device-based assessments that capture multilevel and cross-level behavioural inequalities—linking individual socioeconomic status with regional walkability—remain scarce. We searched PubMed for articles published in English up to August 4, 2025, using combinations of terms related to physical activity inequalities (“physical activit∗“, “distribution∗“, “inequalit∗“, “disparit∗“), regional context (“region∗“, “municipal∗“, “local government∗“, “administrative”), and socioeconomic status (SES) (“socioeconomic status”, “socioeconomic position”, “education∗“, “occupation∗“, “employment∗“, “income”). We also reviewed reference lists and relevant government reports. Existing studies have documented regional disparities (e.g., urban-rural differences) but primarily relied on self-reported data. While some studies have employed device-based measures (e.g., accelerometer or pedometer), none have described nationwide municipal-level distributions based on device-measured activity. Furthermore, despite theoretical and empirical evidence that socioeconomic disparities vary across regional contexts, no nationwide study has examined cross-level interactions between individual SES and the built environment.

**Added value of this study**

To our knowledge, this large-scale descriptive epidemiological study of 1.5 million smartphone users in Japan is the first to quantify nationwide municipality-level inequalities and cross-level interactions between individual-level SES and walkability in step counts. We identified substantial disparities across 951 municipalities, with the maximum difference reaching 3724 steps/day (nearly a 2-fold disparity). Municipalities with higher mean daily steps had greater walkability (r = 0.76) and more equal step-count distributions (Gini coefficients: r = −0.79). However, absolute disparities persisted both between and within municipalities regardless of walkability. At the individual level, significant cross-level interactions existed in all SES domains; however, the pattern of disparities differed across the domains. Notably, step-count differences across walkability strata were prominent for education and income (623–762 steps/day), whereas employment status showed substantial differences between employed and non-employed individuals within a region, reaching 826–1514 steps/day in highly walkable areas.

**Implications of all the available evidence**

Building on global evidence, our findings highlight the need for fine-grained monitoring of physical activity inequalities. Although municipalities with higher walkability showed greater population-level activity, notable gaps persist even in these supportive settings, suggesting that favourable environments alone do not guarantee equitable opportunities for active living. Moreover, vulnerable groups differed by SES and regional context, underscoring the need for targeted, cross-sectoral approaches to address cross-level behavioural inequalities. Integrating high-resolution digital physical-activity data through public-private partnerships can enhance surveillance systems and support data triangulation.

## Introduction

Although the health benefits of physical activity are well established,^1^ global levels of physical inactivity remain high, making its promotion an ongoing public health challenge.^2^ Effective surveillance that identifies high-risk populations is therefore essential for implementing targeted interventions, benchmarking progress, and optimising resource allocation.^3^ While physical activity surveillance has been conducted at global, national, and subnational scales,^2^^,^^4^^,^^5^ such surveillance largely relies on self-reported data, which can capture contextual information but are prone to systematic biases (e.g., recall and social desirability biases^6^). Therefore, surveillance should incorporate device-measured physical activity (e.g., accelerometers and pedometers) alongside self-reports to obtain a more comprehensive picture of population behaviour.^3^

Recently, the widespread adoption of smartphones (global smartphone penetration rate of 78% in 2023^7^) has enabled large-scale, device-based monitoring of physical activity, and smartphone-measured step counts have been validated in both laboratory and free-living settings.^8^^,^^9^ As step counts are straightforward to interpret and have well-documented associations with health outcomes,^10^ they represent a practical and scalable indicator for population surveillance. Moreover, monitoring inequalities across population subgroups is an important aspect of physical activity surveillance, as relying solely on overall averages may mask socioeconomic or demographic disparities.^11^ Surveillance should therefore incorporate inequality monitoring to help ensure that resources are allocated to populations in need.^12^

In many countries, municipalities (the smallest units of local authority, such as cities and towns) are the primary actors in physical activity promotion and locally relevant policy implementation. Understanding geographical distributions of device-measured physical activity at this level is essential for informing evidence-based local strategies for mitigating inequalities. Regional distributions of device-measured physical activity have been reported previously^13^, ^14^, ^15^, ^16^; however, most studies have focused on broader administrative units^13^, ^14^, ^15^ or have lacked nationwide coverage,^16^ resulting in low-resolution descriptions of regional inequalities. For example, studies in the U.S. reported regional physical activity distributions at the state level or multi-state regions,^14^^,^^15^ whereas a study in China examined only selected local government units.^16^ Consequently, nationwide municipality-level distributions of device-measured physical activity remain poorly understood.

Beyond describing these regional inequalities, elucidating activity level disparities between population subgroups and understanding what drives these disparities is crucial.^17^ The built environment and individual socioeconomic status (SES) are important correlates of physical activity,^17^^,^^18^ and previous studies suggest that SES may modify the relationship between the built environment and physical activity.^19^, ^20^, ^21^ However, evidence is limited on whether such disparities vary across regions at a national scale, through cross-level interactions between individual SES and the built environment.

Therefore, we conducted this study using large-scale smartphone-measured step count data from Japan to quantify nationwide municipality-level physical activity inequalities and examine whether SES-related disparities vary based on regional walkability.

## Methods

### Study design and data sources

We conducted a descriptive epidemiological study using nationwide smartphone-measured step count data. We performed municipality- and individual-level analyses to examine disparities in step counts between and within municipalities and cross-level interactions between individual-level SES and regional walkability.

Nationwide data were obtained from the TRIMA app (GeoTechnologies, Inc.), a smartphone application widely used in Japan and other countries (e.g., operating through a similar application, ‘GeoSmile', in the US). TRIMA was launched in October 2020 for the iPhone operating system (iOS) and Android, achieving 22 million downloads and 4 million monthly active users in Japan by April 2025. The app rewards users for activities such as step counts, travel distances (across modes including walking, public transport, and car travel, detected via movement behaviours and trajectories based on the smartphone's global positioning system [GPS]), and completing questionnaires (Appendix Fig. 1). Points can be redeemed for marketplace rewards such as Amazon and Apple gift cards.

All users consented to the app's privacy policy at installation, permitting the use of de-identified data for research. This study was approved by the Ethics Committee of the School of Engineering, The University of Tokyo (approval number: KE25-23).

### Measures

We conducted the analyses using de-identified, individual-level user data collected between January 1 and December 31, 2023, provided by the app operator. The data included smartphone-measured daily step counts, estimated residential locations, and self-reported questionnaire responses. Additionally, we used walkability indices as an environmental exposure metric.

### Step count

The primary outcome was daily steps. Although the app collects step counts through Apple Health (for iOS users), Google Fit (for Android users), and its own accelerometer-based algorithm when users opt not to grant access to these functions, we used step-count data collected only from iOS device users through Apple Health's data-sharing function, as it is a previously validated data source^9^ and explicit third-party data-sharing terms were provided upon request only from Apple Inc. to the app provider. To ensure data validity and plausible ranges, days outside the range of 500–29,999 steps were excluded based on information from previous studies^13^^,^^22^ and empirical data distributions.

### Residential locations and personal characteristics

Users' residential locations were inferred by the provider's residential-location estimation algorithm, which identified the location where users spent the most time between 7:00 pm and 7:00 am over a rolling 7-day window. The algorithm used GPS data of the users' devices, and locations were aggregated to a minimum 125-m grid (with the size varying according to local population distribution) for privacy protection.

Personal characteristics were obtained from self-reported questionnaires administered within the app and included birth year, gender, marital status, presence of children, household size, educational attainment, employment status, and household income. The timing of questionnaire responses varied across users. The step-count data used in this study covered January–December 2023, and users who had completed questionnaires before this period were also included if they had valid step-count records during 2023. Some users had different responses over time; however, response changes were infrequent for the key demographic variables used in this study (0.5–3%), except for household income (11%, which may reflect actual income changes). Therefore, we used the latest available questionnaire data for each user. Age was calculated from birth year and grouped into 20–29, 30–39, 40–49, 50–59, and 60–64 years to account for the non-linear relationship with step counts while maintaining sufficient group sample sizes. Gender, marital status, and presence of children were treated as binary variables (men vs. women, married vs. not married, and having one or more children vs. none). Educational attainment was categorised as university degree or higher, junior college/vocational school, high school or lower, or in school. The original job-related item combined employment status and occupation; for interpretability, responses were recategorised into employment-status groups: regular workers (full-time and government employees, schoolteachers, medical staff, and certified professionals), non-regular workers (contract and part-time), non-workers (homemakers, unemployed, and retirees), executives/managers, self-employed, students, and others (not elsewhere classified). Equivalised household income was calculated by dividing self-reported annual household income by the square root of household size and categorised as ≥5.0, 2.5–4.9, or <2.5 million yen; these cut-points were informed by the approximate sample tertiles and set at interpretable values.

### Walkability

Walkability was defined using a publicly available Japan Postcode-level Walkability Index,^23^ a standardised composite of population density, daily-life facility diversity, and intersection density at the postcode level (Appendix Text 1). Higher values indicate denser, better-connected areas with a greater diversity of facility types (range: −1.85–2.59). The index was validated through its positive association with self-reported walking time and concordance with an address-level walkability index.^23^

### Participant selection

**Fig. 1:**
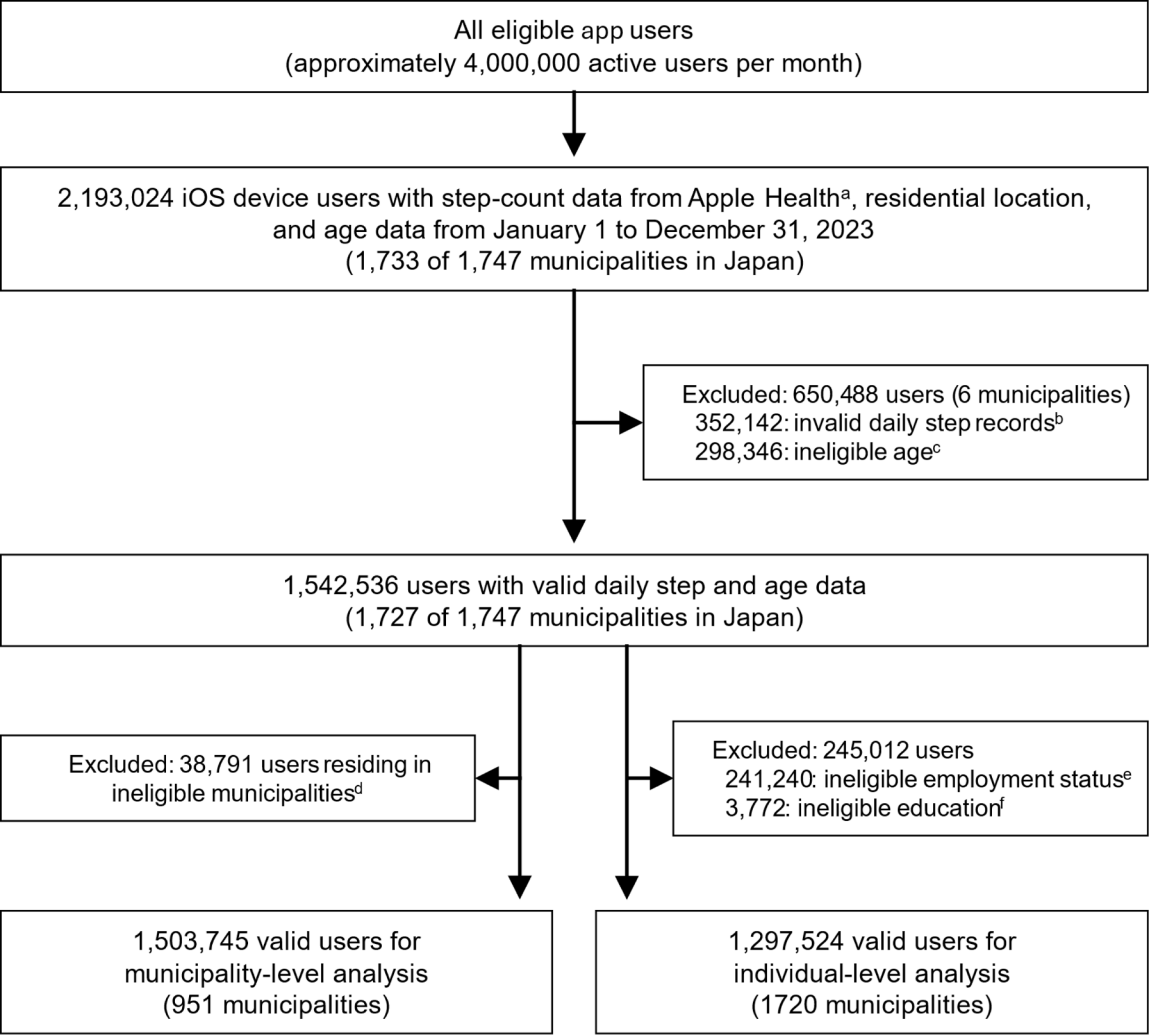
**Flowchart of the inclusion process of study participants**. ^a^ Step-count data were obtained exclusively from iOS device users through Apple Health's data-sharing function; users of Android devices and iOS users who did not grant Apple Health data sharing were not included in the dataset. ^b^ < 10 days of valid daily step records (500–29,999 steps/day). ^c^ Aged <20 years or ≥65 years. ^d^ Municipalities with <100 users in any season (spring: March–May; summer: June–August; autumn: September–November; winter: December–February). ^e^ The original job item included employment status and occupation. To focus on working populations and improve interpretability, we derived an employment-status variable and excluded users classified as student, self-employed, executive/manager, and other (not elsewhere classified). ^f^ Users classified as currently in school were excluded due to a lack of detailed school-related information.

Fig. 1 summarises participant selection. Overall, 2,193,024 users from 1733 of 1747 municipalities provided step count data (318,696,309 person-days), estimated residential locations, and questionnaire data. Eligible users were those who recorded valid daily steps on at least 10 days,^22^ were aged 20–64 years, and had no change in their estimated residential locations at the postcode level.

Different inclusion criteria were applied for the municipality- and individual-level analyses. For the municipality-level analysis, municipalities with at least 100 users per season were included (Appendix Table 1). For the individual-level analyses, we excluded users identified as students or in school (3–7%), and those classified as self-employed (4%), executive/manager (2%) or other (2%) to focus on typical salaried employment.

### Statistical analysis

Group differences in mean daily steps (e.g., men vs. women) were examined using t-tests or analysis of variance on a complete-case basis. As sensitivity analyses, we compared unweighted and weighted mean daily steps to assess whether differences in demographic composition between the analytical sample and the national population affected step-count estimates (details are listed in Appendix Text 2).

### Municipality-level analysis

#### Step count distributions and associations with walkability

For each municipality, we calculated mean daily steps and the Gini coefficient,^24^ a measure of the variation in daily step counts across individuals within each municipality, and mapped their geographical distribution across Japan. We assessed their associations using Pearson's correlation coefficients. Because the Gini coefficient can be influenced by the distributional skewness,^25^ we conducted simulations to assess whether the observed association exceeded mathematically expected values (Appendix Text 3). Additionally, we examined whether municipality-level demographic deviations from the national population were systematically associated with mean daily steps (Appendix Text 4).

We also assessed associations between municipal mean daily steps and municipal walkability, defined as the mean postcode-level walkability index within each municipality. As simple averaging may underestimate walkability by ignoring population distribution, we constructed alternative indicators based on the maximum values and the mean of the top five values within each municipality to assess the robustness.

#### Variations in step count across and within municipalities

We assessed whether between- and within-municipality disparities in step counts varied by municipal walkability. To compare municipalities with similar walkability, we grouped municipalities into five equal-width walkability categories (see Appendix Text 1 for details). Within each category, between-municipal step-count differences were quantified as the interquartile ranges (IQRs) of municipal mean daily steps, and within-municipal step-count differences as the IQRs of users’ mean daily steps.

To examine differences in distributional shape, we estimated municipality-specific distributions of users’ mean daily steps using raw values and values normalised to the municipal mode and compared them using kernel density estimation.

### Individual-level analysis

We examined cross-level interactions between individual-level SES and postcode-level walkability on mean daily steps. The outcome was users’ mean daily steps, and primary exposures were three SES indicators (educational attainment, employment status, and equivalised household income) and walkability. Users were linked to the nearest walkability point within each municipality, and walkability was treated as a continuous variable. Linear and squared terms for walkability were included to capture the non-linear associations observed in preliminary analyses, with improved model fit confirmed using likelihood ratio tests. Cross-level interactions were tested using linear mixed-effects models with random intercepts for municipalities and postcodes, accounting for data clustering. Each SES–walkability interaction (linear and squared terms) was examined separately, adjusting for the other SES variables and covariates (age, marital status, and presence of children). Interaction significance was evaluated using likelihood ratio tests by comparing models with and without the interaction term. Analyses were stratified by gender to account for known gender-based differences in physical activity.^2^^,^^22^ Missing data were handled using multiple imputation (50 datasets) with the Amelia II package in R, assuming data were missing at random, and the imputation model included all variables used in the analyses. Estimates were pooled using the mitml package in R.

For visualisation, adjusted mean daily steps were estimated using the emmeans package in R and pooled across imputations. Walkability was categorised into quintiles, with estimates obtained at the median walkability value within each quintile. Statistical significance was set at p < 0.05. All analyses, processing, and visualisation were performed using Python (version 3.12.4), R (version 4.4.1), and ArcGIS Pro (version 3.2.2, ESRI, Inc.).

### Role of the funding source

The funders of the study had no role in study design, data collection, data analysis, data interpretation, or writing of the report.

## Results

The overall analytical sample comprised 1,542,536 users (261,731,501 person-days) across 1727 municipalities. Sample characteristics are summarised in Table 1. Compared with national statistics, participants were more likely to be younger (52% vs. 38% aged <40 years), women (64% vs. 50%), regularly employed (62% vs. 56%), and to have higher household incomes (54% vs. 35% earning ≥5.0 million yen) (Appendix Table 2). The overall mean daily steps (SD) were 6138 (4651) steps/day, comparable to national survey estimates after adjusting for the reported 12% underestimation in smartphone-based measurements (6901 × 0.88 = 6073 steps/day in NHNS 2022).^9^^,^^26^ After calibrating the sample composition to national population margins, weighted mean daily steps were modestly higher than unweighted estimates across all subgroups (+1 to 365 steps/day; Appendix Text 2 and Appendix Table 3).

**Table 1:** Participant characteristics (total users = 1,542,536).

	Number of users^a^	Mean daily steps (SD)	p values
Age (years), mean (SD)	39.3 (12.1)		
20–29	420,308 (27%)	6278 (7747)	<0.0001^b^
30–39	380,550 (25%)	5926 (7408)	
40–49	364,773 (24%)	5999 (7577)	
50–59	305,006 (20%)	6282 (7943)	
60–64	71,899 (5%)	6593 (8313)	
Gender			
Men	554,725 (36%)	7354 (9000)	<0.0001^c^
Women	987,811 (64%)	5462 (6873)	
Marital status			
Married	831,011 (55%)	5945 (7502)	<0.0001^c^
Not married	693,745 (46%)	6425 (7990)	
Presence of children			
Having one or more children	697,298 (46%)	5756 (7284)	<0.0001^c^
Not having children	827,458 (54%)	6507 (8085)	
Educational attainment			
University graduate or higher	467,363 (31%)	6705 (8200)	<0.0001^b^
Junior college/Vocational school	427,798 (28%)	5716 (7212)	
High school or less	581,359 (38%)	5962 (7623)	
Currently in school	48,236 (3%)	6587 (8010)	
Employment status			
Regular worker	769,894 (50%)	6573 (8128)	<0.0001^b^
Non-regular worker	368,753 (24%)	5702 (7256)	
Non-worker	162,649 (11%)	5057 (6568)	
Executive or manager	37,116 (2%)	6124 (7712)	
Self-employed	66,359 (4%)	5764 (7351)	
Student	104,565 (7%)	6606 (7996)	
Other	33,200 (2%)	5799 (7363)	
Equivalised household income^d^, Japanese yen			
≥5.0 million yen	236,355 (19%)	6686 (8208)	<0.0001^b^
2.5–4.9 million yen	630,439 (51%)	6237 (7802)	
<2.5 million yen	375,312 (30%)	5777 (7380)	
Walkability index^e^, mean (SD)	0.9 (0.7)		
C5: 1.48–2.59	308,575 (20%)	5336 (6951)	<0.0001^b^
C4: 1.10–1.47	308,442 (20%)	5626 (7236)	
C3: 0.78–1.09	308,543 (20%)	5985 (7564)	
C2: 0.37–0.77	308,536 (20%)	6562 (8063)	
C1: −1.85 to 0.36	308,440 (20%)	7177 (8578)	

Users aged 20–64 years with valid step count records (≥10 days with 500–29,999 steps/day) were included.

a Values are presented as n (%) or mean (SD). User counts vary due to missing responses.

b Differences in mean daily steps across demographic groups were analysed using ANOVA.

c Differences in mean daily steps across demographic groups were analysed using t-tests.

d Calculated by dividing annual household income by the square root of household size.

e Defined using the Japan Postcode-level Walkability Index.^23^ Its values were categorised into quintiles.

We conducted municipality- and individual-level analyses; Appendix Table 4 summarises characteristics for each analytic sample.

For the municipality-level analysis, we included 951 municipalities with at least 100 users (1,503,745 users), covering 96% of the Japanese population in the targeted municipalities. Mean daily steps among these users was 6163 (4657) steps/day.

**Fig. 2:**
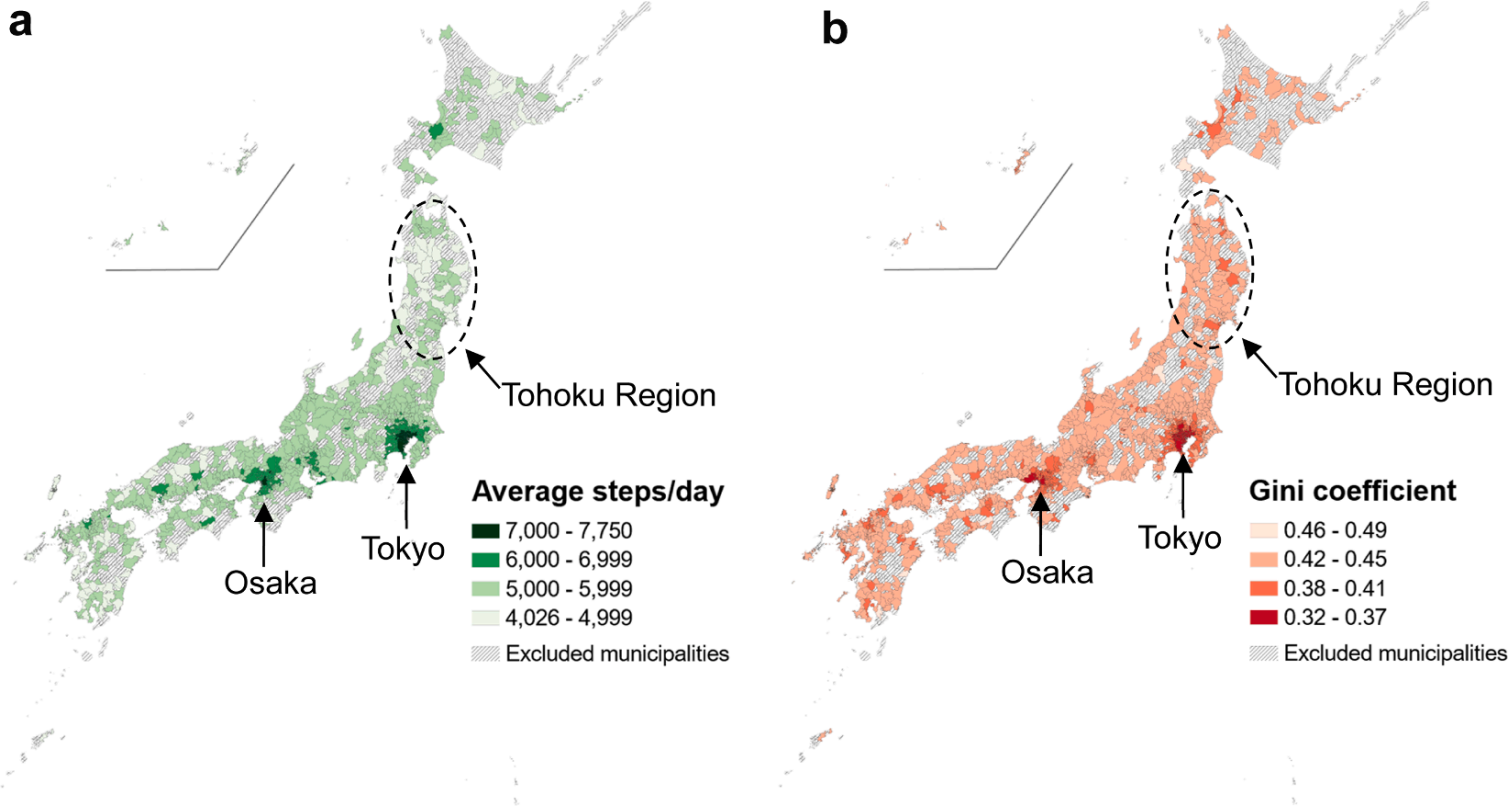
**Geographical distribution of (a) municipal mean daily steps and (b) Gini coefficients of step counts**. This analysis included 951 of the 1747 municipalities in Japan. Shaded areas indicate excluded municipalities, defined as those with <100 users in any season (spring: March–May; summer: June–August; autumn: September–November; winter: December–February). The locations of Tokyo, Osaka, and the Tohoku region are indicated in the figures.

Across municipalities, the mean daily steps (SDs) ranged from 4026 (3405) to 7750 (8591) steps/day, a difference of 3724 steps/day. There was no clear evidence that municipality-level demographic deviations between the study sample and the national population were systematically associated with mean daily steps except for age; however, this association was attenuated after accounting for walkability (Appendix Text 4, Appendix Table 5, and Appendix Fig. 2). Fig. 2 shows the geographical distribution of municipal mean daily steps and Gini coefficients. Appendix Table 6 ranks the top and bottom municipalities according to mean daily steps. Urbanised municipalities, particularly around Tokyo and Osaka, showed higher mean daily steps and more equal step distributions, whereas rural regions such as Tohoku (northeastern Japan) generally showed lower means and greater variability. Municipal mean daily steps correlated positively with municipal walkability (r = 0.76) and negatively with Gini coefficients (r = −0.79) (Appendix Fig. 3). Appendix Fig. 4 shows the nationwide maps of municipal mean daily steps (before applying the ≥100-user criterion) and walkability. Sensitivity analyses using alternative walkability definitions yielded similar results (Appendix Fig. 5). Simulated step-count distributions did not produce a stronger negative correlation between mean daily steps and Gini coefficients than that observed empirically (Appendix Table 7, Appendix Fig. 6).

**Fig. 3:**
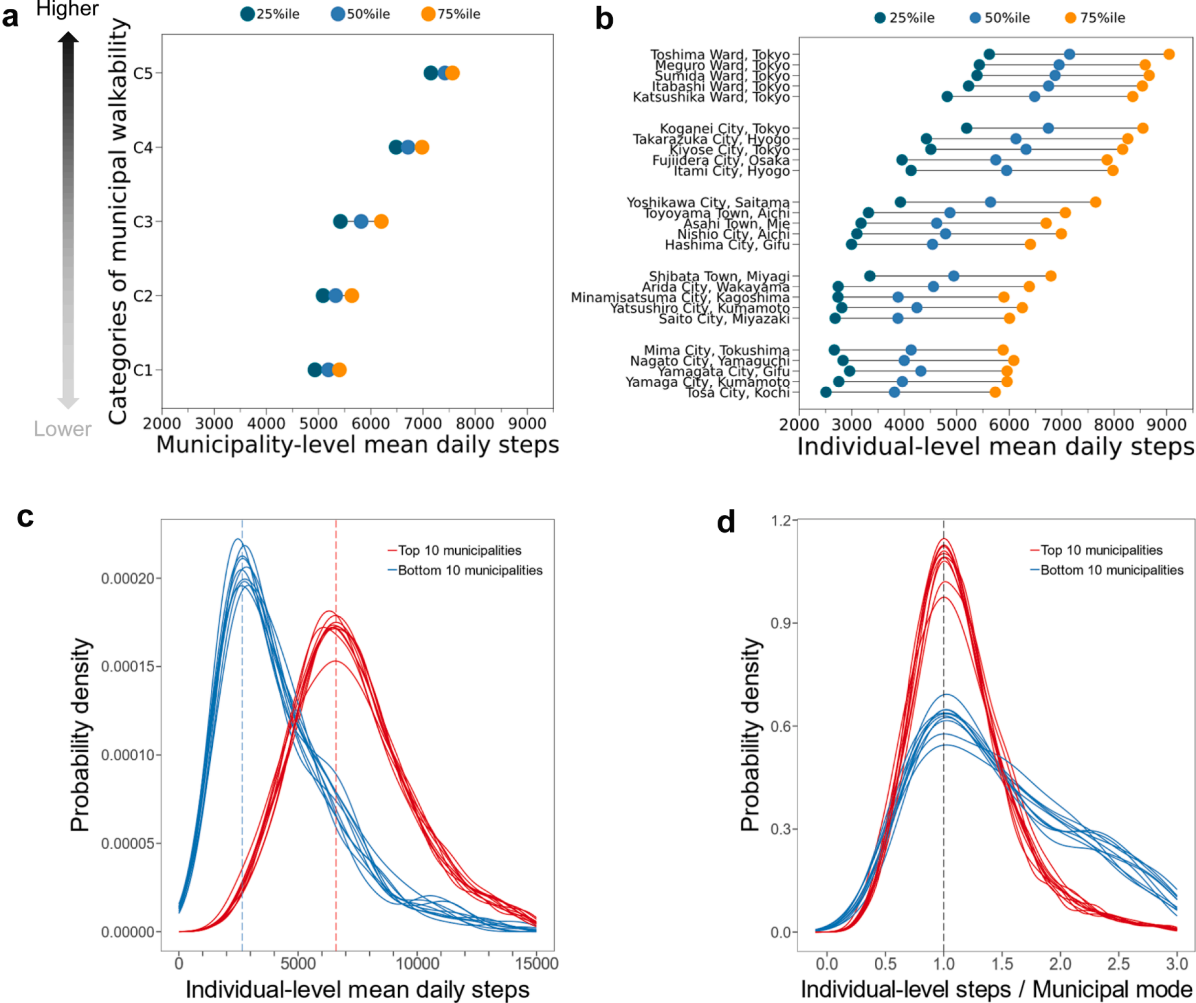
**Variability and distributions of mean daily steps between and within municipalities**. Panel (a) shows the interquartile ranges (IQRs) of municipality-level mean daily steps across 951 municipalities, stratified by municipal walkability. Panel (b) shows the IQRs of individual-level mean daily steps within municipalities, based on five randomly selected municipalities per walkability category (total n = 25, labelled as “City/Town/Ward, Prefecture”). Municipal walkability was defined as the municipality-level mean of the Japan Postcode-level Walkability Index,^23^ and categorised into five equal-width groups: C1 (−1.11 to −0.44), C2 (−0.45 to 0.21), C3 (0.22 to 0.88), C4 (0.89 to 1.55), and C5 (1.56 to 2.24). Panels (c) and (d) show distributions of individual-level mean daily steps in the top 10 (red) and bottom 10 (blue) municipalities ranked by municipal mean daily steps. Panel (c) shows raw distributions, with red and blue dashed lines indicating the mode for Toshima Ward, Tokyo (the highest-step municipality) and Kobayashi City, Miyazaki (the lowest-step municipality), respectively. Panel (d) shows mode-normalised distributions, with the municipal mode indicated by the black dashed line.

Fig. 3 shows between- and within-municipality variability in mean daily steps. Between-municipal IQRs of mean daily steps (Fig. 3a) and within-municipal IQRs of individual-level mean daily steps (Fig. 3b) were both stable across walkability categories, whereas within-municipal IQRs were consistently larger. Similar results were observed using alternative walkability definitions (Appendix Table 8). Comparing the distributions of individual-level mean daily steps across top and bottom 10 municipalities ranked by their mean daily steps, low-step municipalities showed lower modes, more right-skewed distributions (Fig. 3c), greater relative variance (Fig. 3d), and higher Gini coefficients (0.41–0.44 vs. 0.33–0.35; Appendix Table 6). Sensitivity analyses of municipalities with ≥1000 users (Appendix Fig. 7) and stratified by municipal walkability (Appendix Fig. 8) yielded consistent results.

For the individual-level analysis, 1,297,524 users with a mean step count of 6164 (4668) steps/day were included. Household income had a 17% missing rate owing to self-reported “unknown” responses (Appendix Table 9).

**Fig. 4:**
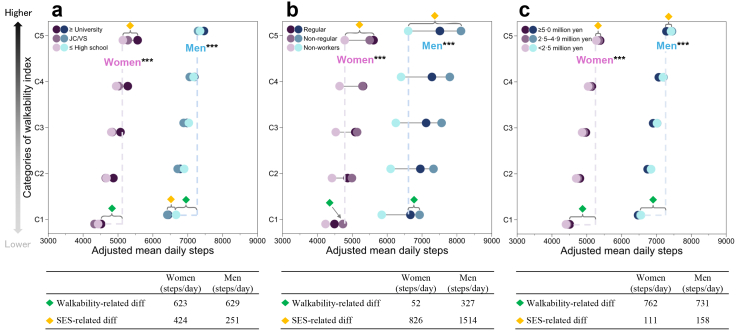
**Cross-level inequalities in mean daily steps based on individual-level socioeconomic status (SES) and walkability**. These figures show model-adjusted mean daily steps estimated from linear mixed-effects models, illustrating the cross-level interaction between individual SES and walkability, stratified by gender. Walkability was defined using the Japan Postcode-level Walkability Index.^23^ For visualisation, the walkability index was categorised into quintiles: C1 (−1.85 to 0.36), C2 (0.37 to 0.76), C3 (0.77 to 1.09), C4 (1.10 to 1.47), and C5 (1.48 to 2.59). Adjusted mean daily steps were estimated at the median walkability value within each quintile. Panels display the interactions between walkability and each SES indicator: (a) educational attainment, (b) employment status, and (c) equivalised household income (Japanese yen). Models were adjusted for age, marital status, presence of children, and the other SES indicators not used in the interaction term. ≥ University: university degree or higher. JC/VS: Junior College or Vocational School. ≤ High school: high school or less. Regular: regular worker. Non-regular: non-regular worker. p for interaction: ∗ ≤0.05, ∗∗ ≤0.01, and ∗∗∗ ≤0.001. Walkability-related (between-area) diff: the absolute difference between the lowest-step SES subgroup in C5 (the highest walkability quintile) and the highest-step SES subgroup in C1 (the lowest walkability quintile), representing a conservative (minimum) estimate of the step-count gap associated with walkability. SES-related (within-area) diff: the largest difference in estimated mean daily steps among SES subgroups within any single walkability quintile.

Fig. 4 shows the model-adjusted mean daily steps across SES subgroups. Appendix Tables 10 and 11 detail the model estimates. All models showed significant cross-level interactions, suggesting that the relationships between step counts and SES varied by walkability, with inconsistent patterns observed by gender. For example, regarding educational attainment, women with a university degree or higher consistently recorded more steps than other educational groups, with larger differences in highly walkable areas. Among men, the most active subgroup shifted from those with a high school education or lower in the least walkable areas (C1) to those with a university degree or higher in the most walkable areas (C5), although step differences remained relatively stable across walkability levels (Fig. 4a).

To evaluate the disparities related to SES and walkability in step counts, we contrasted two complementary measures: “SES-related (within-area) differences”, defined as the largest step-count differences between SES subgroups within the same walkability stratum (yellow markers in Fig. 4), and “walkability-related (between-area) differences”, defined as the differences between the highest-step SES subgroup in the least walkable area (C1) and the lowest-step SES subgroup in the most walkable area (C5; green markers in Fig. 4). This represents a conservative (minimum) estimate of the walkability-related differences isolated from SES-specific susceptibility to walkability, because selecting the maximum or minimum C5–C1 difference calculated within each SES subgroup would otherwise conflate the walkability-related difference with this susceptibility under cross-level interactions. Overall, step-count differences across walkability strata (walkability-related differences) were 623–762 steps/day for education and income (Fig. 4a and c), whereas for employment status, they were small (327 and 52 steps/day for men and women, respectively; Fig. 4b). In contrast, the step-count differences among SES subgroups within a walkability stratum (SES-related differences) were modest for education and income (up to 424 steps/day, Fig. 4a and c) but substantially larger for employment status, reaching 1514 and 826 steps/day for men and women in the most walkable areas (Fig. 4b). The details of the step count estimates are listed in Appendix Table 12.

## Discussion

To our knowledge, this is the first study using nationwide smartphone data to examine municipality-level step-count distributions and cross-level inequalities between individual-level SES and walkability. We identified three key findings. First, substantial disparities existed across municipalities, with step-count differences of up to 3724 steps/day. Second, municipalities with lower mean daily steps tended to be less walkable and exhibited greater inequality. Third, at the individual level, SES and walkability showed significant cross-level interactions, with lower-step subgroups differing across walkability levels. Importantly, the magnitude and patterns of disparities varied according to the SES dimension: walkability was associated with prominent step-count differences for education and income (623–762 steps/day), whereas employment status itself accounted for substantial disparities (up to 1514 steps/day within walkability strata).

Geographical disparities in physical activity have been documented across and within countries.^2^^,^^4^^,^^5^^,^^13^, ^14^, ^15^, ^16^^,^^22^ Our findings indicate that step-count disparities across Japanese municipalities are comparable to, or greater than, previously reported findings,^13^, ^14^, ^15^^,^^22^ and highly walkable municipalities showed higher step counts and lower inequality, consistent with the existing literature.^22^^,^^27^ However, absolute within-municipality gaps (IQRs) persisted across walkability levels, implying that favourable built environments alone were not necessarily accompanied by smaller absolute disparities.

The relationship between SES and step counts varied by walkability. Consistent with previous findings with smaller samples and limited geographical coverage,^19^, ^20^, ^21^ individuals with a university degree or higher recorded more steps in highly walkable areas than other educational groups, suggesting that this group might disproportionately benefit from walkable environments, potentially through the adoption of a more active lifestyle.^28^ In contrast, income-related associations were relatively stable across walkability levels, unlike previous reports.^19^^,^^21^ This discrepancy may reflect methodological differences (e.g., neighbourhood-level income and self-reported data in prior studies vs. household income and device-measured data in ours); further nationwide studies are warranted to clarify regional variations in income–activity associations.

Although step-count disparities for education and income showed similar patterns, with prominent differences across walkability strata, employment status played a distinct role. Disparities by employment status (employed or non-employed) were larger than education- and income-related disparities within a single walkability stratum, and these gaps were more pronounced in highly walkable areas. This pattern may be related to interactions between employment-related behaviours (e.g., active commuting) and the built environment.^18^^,^^21^^,^^29^ Consequently, improving walkability alone may inadvertently widen existing inequalities; therefore, careful monitoring of disparities is needed.

Our findings have several policy implications. First, inequality monitoring should accompany physical activity surveillance,^12^ as some groups (e.g., unemployed individuals) consistently recorded lower steps regardless of walkability. Identifying who benefits and who is left behind would support more targeted and equitable interventions.^11^^,^^12^ Second, addressing physical activity inequalities requires attention to multilevel drivers.^12^ While walkable environments may improve population-level physical activity, modifying the built environment is resource-intensive. Moreover, absolute disparities persisted even within highly walkable areas and high-risk (low-step) groups varied by regional context, suggesting that integrated approaches targeting both regional and individual factors are needed. Lastly, digital physical-activity data offer unprecedented opportunities for continuous, fine-grained monitoring beyond traditional surveys, and for accelerating data triangulation on population behaviour. Despite challenges such as privacy protection and technological inequalities,^30^ cross-sectoral collaborations among government, industry, and academia could facilitate the use of large-scale, high-resolution data to inform evidence-based policymaking.^3^

Despite strengths, such as utilising large-scale, nationwide device-based step count data, this study has several limitations. First, participants differed demographically from the national population, limiting generalisability. The app is advertised on social media platforms and may overrepresent active social media users and those willing to share personal data. Additionally, women, higher-education, and higher-income groups are more likely to use iPhone^31^ and adopt mHealth applications, whereas older adults are less likely,^32^ which may have contributed to the skewed sample composition. However, a sensitivity analysis using national survey weights showed only modest differences in mean daily steps, and the overall step counts in the main analysis were comparable to national survey estimates after accounting for an estimated 12% smartphone-measured underestimation.^9^ Moreover, the association between age-related demographic deviation and mean daily steps at the municipality level was attenuated after accounting for walkability (Appendix Fig. 2), suggesting that the observed disparities were not fully attributable to selection bias. Second, smartphone-measured step counts may underestimate actual steps, with the degree of underestimation likely varying by personal characteristics.^9^ Compared with national surveys, step counts were comparable for men (7354 vs. 7323 steps/day in NHNS 2022) but lower for women (5462 vs. 6536 steps/day),^26^ likely reflecting gender-related differences in phone-carrying behaviour.^9^ Measurement error may also vary by other factors such as occupational context, regional differences in occupational composition, seasonal variations in clothing (the number of pockets available for carrying a smartphone might vary across seasons), and where people primarily spend their time (e.g., at home, where phones may be carried less frequently). Consequently, some observed step-count variation may partly reflect differential measurement error. Future studies should therefore incorporate information on device-carrying behaviours. Third, municipalities with fewer than 100 users were excluded from municipality-level analyses, although population coverage remained high (96%). Fourth, the walkability index used in this study was aggregated at the postcode level and may not accurately reflect the built environment within each individual's activity space. Future studies with greater computational resources are encouraged to incorporate activity-space-based walkability measures leveraging GPS data, which would further refine the characterisation of environmental exposure. Lastly, individual-level models did not adjust for other relevant factors, such as health status,^17^ which could provide further insights if available.

In conclusion, this nationwide analysis demonstrated marked inequalities in daily activity levels, with substantial variations across and within municipalities. Although higher walkability was associated with greater population-level activity, notable gaps persisted even in highly walkable settings, indicating that supportive environments alone do not ensure equitable opportunities for active living. Cross-level inequalities, arising from interactions between individual SES and walkability, underscore the importance of monitoring distributional patterns in addition to average activity levels. These findings support nationwide, device-based surveillance to identify populations vulnerable to inactivity and the development of multilevel, cross-sectoral strategies to promote physical activity across diverse population groups.

## Contributors

KA and MK developed the study design and protocol. TY and KH received the de-identified raw data from the provider, and KA and MK had the access to the stored data. KA conducted the primary analyses and drafted the manuscript. All authors provided critical input during the writing and revision of the paper, and accept responsibility for the decision to submit the manuscript. MK attests that all listed authors meet the authorship criteria and that no others meeting the criteria have been omitted. KA and MK have directly accessed and verified the data reported in the manuscript.

## Data sharing statement

The de-identified data were provided by GeoTechnologies, Inc. Under the research agreement, the authors were granted access to de-identified data and are prohibited from sharing any individual-level data. The raw data is not publicly available; interested researchers may purchase de-identified data by contacting the company. Aggregated municipality-level data are available here: https://doi.org/10.82337/1000011.

## Editor note

The Lancet Group takes a neutral position with respect to territorial claims in published maps and institutional affiliations.

## Declaration of generative AI and AI-assisted technologies in the manuscript preparation process

During the preparation of this work, the authors used ChatGPT to improve the readability and language of the manuscript. After using this tool, the authors reviewed and edited the content as needed and take full responsibility for the content of the published article.

## Declaration of interests

The authors have no competing interests to disclose.
