# Supplementary material for: Cross-level regional and socioeconomic inequalities in step counts: a descriptive epidemiological investigation of 1.5 million Japanese smartphone users
Source: Lancet Reg Health West Pac. 2026 Jun 19;72:101901. doi: 10.1016/j.lanwpc.2026.101901 (PMC13478815; doi:10.1016/j.lanwpc.2026.101901)
Supplement: Supplementary file 1 [file mmc1.pdf]

Supplementary appendix to the article

Cross-level Regional and Socioeconomic Inequalities in Step Counts: A Descriptive Epidemiological Investigation of 1·5 Million Japanese Smartphone Users

Contents

Appendix Text 1. Details of the walkability index and its categorisation 2

Appendix Text 2. Sensitivity analysis: comparison of unweighted and weighted demographic composition and step-count estimates 2

Appendix Text 3. Simulation methods for the association between municipal mean daily steps and the Gini coefficient 3

Appendix Text 4. Municipality-level demographic deviations and their association with mean daily steps 4

Appendix Text 5. Residential location estimation and its association with step-count estimates 4

Appendix Text 6. Association between questionnaire response timing and step counts 5

Appendix Table 1. Comparison of 95% confidence interval widths for mean daily steps between TRIMA and NHNS data 6

Appendix Table 2. Demographic characteristics of TRIMA users compared with national statistics in Japan 7

Appendix Table 3. Unweighted and weighted mean daily steps by participant characteristics (N = 1,242,106 users) 8

Appendix Table 4. Characteristics of participants in the municipality-level and individual-level analyses 9

Appendix Table 5. Municipality-level demographic proportions of TRIMA users and the national population 10

Appendix Table 6. Municipality-level summary statistics for the number of users, mean daily steps, and Gini coefficients 11

Appendix Table 7. Simulation results for hypothetical users' step counts and correlations between simulated municipal mean daily steps and Gini coefficients 11

Appendix Table 8. Summary of users, municipalities, and step-count variability by municipal walkability definition 12

Appendix Table 9. Proportions of missing data for variables included in multiple imputation 13

Appendix Table 10. Results of linear mixed-effects models for mean daily steps in men 14

Appendix Table 11. Results of linear mixed-effects models for mean daily steps in women 15

Appendix Table 12. Model-adjusted mean daily steps by socioeconomic status and walkability categories, stratified by gender .. 16

Appendix Figure 1. Screenshots of the TRIMA app 17

Appendix Figure 2. Association between municipality-level demographic deviations and mean daily steps, before and after adjustment for walkability 18

Appendix Figure 3. Associations of municipal mean daily steps with municipal walkability and Gini coefficients 19

Appendix Figure 4. Nationwide geographical distribution of municipal mean daily steps and walkability 19

Appendix Figure 5. Association between municipal mean daily steps and alternative definitions of municipal walkability 20

Appendix Figure 6. Simulated distributions of user- and municipality-level step counts from 5,000 simulations 21

Appendix Figure 7. Within-municipality distributions of individual-level mean daily steps (municipalities with ≥1,000 users).... 22

Appendix Figure 8. Within-municipality distributions of individual-level mean daily steps based on municipal walkability categories 22

References 23

Appendix Text 1. Details of the walkability index and its categorisation

Walkability was defined using a publicly available Japan Postcode-level Walkability Index developed by Tanimoto et al.[1] Postcodes in Japan denote postal delivery areas rather than administrative units, and their geographic size varies across regions. Based on its dataset-derived estimates, they range from approximately 0·1 km² in urban areas to over 100 km² in rural areas.

The index comprised three components within a 1,000-m road distance from the most populated 250-m grid per postcode: (1) population density, (2) the count of predefined daily-life facility types, and (3) intersection density. The facility-type component measures the diversity of nearby daily-life facilities rather than their total count, defined as how many of 18 predefined categories are present (range: 0–18). The 18 facility types are: convenience stores, supermarkets/department stores, other food stores, bookstores, pharmacies, government offices, community centres, libraries, cafés/coffee shops, fast-food restaurants, other restaurants, gyms/fitness clubs, urban parks, post offices, banks, medical facilities, dry cleaners, and barbershops/hair salons.

Each component was square root–transformed and standardised, and the index was calculated as the mean of the three components. Higher values indicate denser, better-connected areas with a greater diversity of facility types (range: -1·85–2·59). The index was validated through its positive association with self-reported walking time and concordance with an address-level walkability index.[1]

For the municipality-level analysis, we grouped municipalities into five equal-width walkability categories (i.e., categories defined by dividing the walkability index range into five intervals of equal width), because quantile-based categorisation would produce unequal index ranges across categories (Appendix Table 8), undermining the comparability of municipalities, particularly in higher-walkability categories.

Appendix Text 2. Sensitivity analysis: comparison of unweighted and weighted demographic composition and step-count estimates

We conducted a sensitivity analysis to assess whether differences in demographic composition between the analytical sample and the national population affected step-count estimates. Survey weights were calibrated to national population margins for variables showing substantial proportion differences in Appendix Table 2, including age (five categories: 20–29, 30–39, 40–49, 50–59, and 60–64 years), gender (men and women), and household income (three categories: <3·0, 3·0–4·9, and ≥5·0 million yen). Target distributions were obtained from the 2020 Population Census (age and gender) and the 2018 Housing and Land Survey (household income), accessed via the Portal Site of Official Statistics of Japan (e-Stat). Because these variables were collected in separate national surveys, cross-tabulated population counts were not available; iterative proportional fitting was therefore used, as it requires only marginal distributions. From the overall analytical sample (n = 1,542,536), users with missing household income were excluded (n = 300,430); the remaining 1,242,106 users were included. Educational attainment and employment status were not used for weight calibration and weighted estimates for these variables reflect the indirect effect of age, gender, and income adjustment. The results before and after weighting are listed in the Appendix Table 3.

After weighting, the demographic composition of the analytical sample closely matched the national population for all three calibration variables. For educational attainment and employment status, which were not used for calibration, the weighted proportions also shifted towards the national population. The overall weighted mean daily steps were 6,425 (SD 4,896) steps/day, compared with 6,195 (SD 4,686) steps/day before weighting—a difference of 230 steps/day. Across demographic subgroups, the step-count change ranged from 1 to 365 steps/day, with no subgroup showing a disproportionately large change. These results suggest that the observed step-count estimates remained largely consistent even after adjusting for the demographic imbalances between the analytical sample and the national population.

Appendix Text 3. Simulation methods for the association between municipal mean daily steps and the Gini coefficient

We conducted a simulation analysis to evaluate whether the observed correlations between municipal mean daily steps and the Gini coefficients[2] were stronger than mathematically expected correlations. The simulation was designed to generate a dataset comprising 10,000 hypothetical users, distributed across 100 hypothetical municipalities. Each simulation proceeded as follows. First, we generated a series of daily steps for each of the 10,000 hypothetical users. Each user was randomly assigned a number of days with recorded steps, ranging from 10 to 365 days. Daily step counts for each day were drawn from one of three probability distributions: normal, log-normal, or gamma distributions. The parameters of these distributions were calibrated to match the grand mean (m: 6,163) and standard deviation (s: 4,657) observed in the empirical samples of the municipality-level analysis. For the normal distribution, daily steps were drawn from the $steps \sim N(m, s^2)$. For the log-normal distribution, daily steps were drawn from $steps \sim Lognormal(\mu_{log}, \sigma^2_{log})$, where the log-scale mean (μ_{log}) and variance ($\sigma^2{}_{log}$) were derived from the empirical mean (m) and standard deviation (s) as follows:

$$\mu_{log} = \ln\left(\frac{m}{\sqrt{1 + \frac{s^2}{m^2}}}\right)$$

$$\sigma^2_{log} = \ln\left(1 + \frac{s^2}{m^2}\right)$$

For the gamma distribution, daily steps were drawn from $steps \sim Gamma(\alpha, \theta)$, with the shape ($\alpha$) and scale ($\theta$) parameters calculated from the empirical mean (m) and standard deviation (s) using the following equation:

$$\alpha = \frac{m^2}{s^2}$$

$$\theta = \frac{s^2}{m}$$

Daily step counts outside the range of 500–29,999 were excluded, and data generation continued until 10,000 users with valid daily step records were obtained. These users were then evenly allocated across 100 hypothetical municipalities (100 users per municipality). For each municipality, we calculated the mean daily steps and the Gini coefficient of step counts, and subsequently computed the correlation coefficient between these two municipal-level statistics. The entire procedure was repeated 5,000 times. The simulated distributions of step counts and the resulting associations between municipal mean daily steps and the Gini coefficient for each probability distribution are presented in Appendix Table 7 and Appendix Figure 6.

Appendix Text 4. Municipality-level demographic deviations and their association with mean daily steps

This analysis assessed (1) the uniformity of demographic deviations between TRIMA users and the national population across municipalities and (2) whether these deviations were associated with municipality-level mean daily steps.

For each of the 951 municipalities (939 for household income), we calculated the proportion of five demographic subgroups (age 20–49 years, women, university education or higher, regular employee, and household income ≥5·0 million yen) among TRIMA users and the national population, respectively. The deviation was defined as the TRIMA user proportion minus the national population proportion within each municipality. National population data were obtained from the 2020 Population Census for age, gender, education, and employment status, and from the 2018 Housing and Land Survey for household income, both accessed via the Portal Site of Official Statistics of Japan (e-Stat). Demographic deviations existed for all five characteristics across municipalities, but were relatively consistent, as indicated by narrow interquartile ranges (see Appendix Table 5).

To examine whether these deviations were associated with municipality-level mean daily steps, we visualised the association between each municipality's demographic deviation and its mean daily steps; Pearson's correlation coefficients were also calculated (Appendix Figure 2). Because both demographic deviations and mean steps may be confounded by urbanicity—rural municipalities tend to have older populations as well as lower walkability (thus a higher age-proportion deviation might correlate with lower step counts)—we additionally plotted walkability-adjusted mean daily steps. Walkability-adjusted mean daily steps were calculated by ordinary least squares regression, utilising the municipal mean of the Japan Postcode-level Walkability Index.[1]

Overall, the scatter plots did not reveal a clear pattern whereby municipalities with larger deviations systematically had higher or lower step counts, except for age (Appendix Figure 2). The deviation of age proportion showed a negative correlation with mean daily steps ($r = -0{\cdot}70$); however, this association was attenuated after adjusting for walkability ($r = -0{\cdot}29$). This suggests that differences in the demographic composition of TRIMA users across municipalities are unlikely to have substantially biased municipality-level step-count estimates.

Appendix Text 5. Residential location estimation and its association with step-count estimates

To assess the plausibility of the residential location estimation, we compared GPS-inferred residential prefectures with self-reported prefectures from the in-app questionnaire (as self-reported addresses were only available at the prefecture level) for the overall analytical sample (n = 1,542,536); the concordance rate was 93%. The 7% discordance may partly reflect factors such as relocation after answering the questionnaire. Restricting the analysis to prefecture-concordant users yielded no meaningful differences in overall mean daily steps (6,138 vs. 6,123 steps/day) or the maximum municipality-level step-count difference (3,724 vs. 3,708 steps/day), suggesting that this discordance is unlikely to have materially affected the main findings.

Appendix Text 6. Association between questionnaire response timing and step counts

Questionnaire responses were collected within the app, and the timing of responses varied across users. The step-count data used in this study covered January–December 2023, and users who had completed questionnaires before this period were also included if they had valid step-count records during 2023. Therefore, we evaluated whether questionnaire response timing affected step counts. After adjusting for the covariates used in the individual-level analysis, users who responded during the step-count observation period recorded 335 more steps/day than those who responded before it. This difference may reflect unmeasured factors; for example, users who installed the app during the step-count observation period may have had greater motivation to earn rewards through in-app activities such as step counts, compared with those who installed it earlier and whose motivation may have diminished over time. However, this difference was smaller than the main step-count differences examined (e.g., 700–1,500 steps/day across SES and walkability strata). Also, across quintile-based walkability strata, both the proportion of users who responded during 2023 (approximately 55%) and the step-count difference (325 to 349 steps/day) were consistent, suggesting that the observed primary findings cannot be attributed solely to differences in questionnaire timing.

Appendix Table 1. Comparison of 95% confidence interval widths for mean daily steps between TRIMA and NHNS data

Data source[a]	Inclusion criteria[b]	Participants, n (%)	Municipalities/prefectures, n (%)	Maximum 95% CI[c] width (steps/day)
TRIMA	Unrestricted	1542536 (100)	1727 (100)	22033
	Municipalities with ≥50 users	1526194 (99)	1162 (67)	2512
	Municipalities with ≥100 users	1503745 (97)	951 (55)	1729
	Municipalities with ≥500 users	1325112 (86)	434 (25)	875
	Municipalities with ≥1000 users	1136001 (74)	244 (14)	875
NHNS 2012				
Male		7367	47	3187
Female		8618	47	1789
NHNS 2016				
Male		5598	46	2870
Female		6554	46	2844

[a] Values represent municipality-level data from the TRIMA app and prefecture-level data from the National Health and Nutrition Survey in Japan (NHNS).[3,4] Eligible TRIMA users were aged 20–64 years and recorded 500–29,999 steps/day on at least 10 days.

[b] For the TRIMA data, municipalities were included only if they met the specified user-count threshold in every season (spring: March–May; summer: June–August; autumn: September–November; winter: December–February).

[c] CI: Confidence interval. Values represent the maximum width of the 95% confidence interval for municipality- or prefecture-level mean daily steps.

The threshold of ≥100 users per season in the municipality-level analysis was selected so that the widest 95% CI of municipality-level mean daily steps did not exceed the prefectural-level 95% CI from the NHNS.

Appendix Table 2. Demographic characteristics of TRIMA users compared with national statistics in Japan

	National statistics, n (%)	TRIMA users[a], n (%)	P values[b]
Age[cd]	67305324	1542536	
20–29	11963270 (18)	420308 (27)	<0·0001
30–39	13796161 (20)	380550 (25)	
40–49	17941370 (27)	364773 (24)	
50–59	16307333 (24)	305006 (20)	
60–64	7297190 (11)	71899 (5)	
Gender[cd]	67305324	1542536	
Women	33431837 (50)	987811 (64)	<0·0001
Educational attainment[ce]	57321899	1524756	
University graduate or higher	17683248 (31)	467363 (31)	<0·0001
Junior college / Vocational school	11610422 (20)	427798 (28)	
High school or less	25720992 (45)	581359 (38)	
Currently in school	2307237 (4)	48236 (3)	
Employment status[f]	54857767	1242122	
Regular worker	30796831 (56)	769894 (62)	<0·0001
Non-regular worker	16145390 (29)	368753 (30)	
Executive	3134199 (6)	37116 (3)	
Self-employed	4781347 (9)	66359 (5)	
Household income[g] (Japanese Yen)	49572500	1242106	
≥5·0 million yen	17278000 (35)	670453 (54)	<0·0001
3·0–4·9 million yen	13903300 (28)	329172 (27)	
<3·0 million yen	18391200 (37)	242481 (20)	

[a] All users aged 20–64 years with valid step count records (≥10 days with 500–29,999 steps/day) were included. However, educational attainment, employment status, and household income were limited to the categories shown in the table; therefore, these variables do not cover the entire analytical sample.

[b] Chi-squared tests were used.

[c] The age range in the national statistics was restricted to between 20–64 years.

Japanese national statistics sources:

[d] Portal site of official statistics of Japan (e-Stat), Population census 2020, Basic complete tabulation on population and house-hold, Table number 2–7.

[e] Portal site of official statistics of Japan (e-Stat), Population census 2020, Basic complete tabulation on labor force, Table number 11–2.

[f] Portal site of official statistics of Japan (e-Stat), Population census 2020, Basic complete tabulation on labor force, Table number 5–3.

[g] Portal site of official statistics of Japan (e-Stat), Housing and land survey 2018, Basic tabulation on dwelling and households, Table number 44–4.

Appendix Table 3. Unweighted and weighted mean daily steps by participant characteristics (N = 1,242,106 users)

	Users, n	Unweighted, %	Weighted, %	National population, %	Unweighted mean steps (SD)	Weighted mean steps (SD)	Difference[a]
Overall	1242106	100	100	NA	6195 (4686)	6425 (4896)	230
Age, years							
20–29	285598	23	18	18	6308 (4559)	6469 (4669)	161
30–39	326138	26	20	20	5987 (4463)	6206 (4642)	219
40–49	310076	25	27	27	6075 (4666)	6282 (4835)	207
50–59	258623	21	24	24	6353 (4900)	6504 (5037)	151
60–64	61671	5	11	11	6662 (5109)	6851 (5260)	189
Gender							
Men	486406	39	50	50	7370 (5185)	7379 (5304)	9
Women	755700	61	50	50	5428 (4154)	5449 (4222)	21
Household incomed, Japanese yen							
≥5·0 million yen	670453	54	35	35	6268 (4665)	6573 (4842)	305
3·0–4·9 million yen	329172	27	28	28	6124 (4705)	6403 (4913)	279
<3·0 million yen	242481	20	37	37	6068 (4721)	6285 (4933)	217
Educational attainment							
University graduate or higher	402577	32	29	31	6760 (4743)	7038 (4964)	278
Junior college / Vocational school	349275	28	26	20	5750 (4419)	5983 (4618)	233
High school or less	470584	38	43	45	6008 (4786)	6250 (4977)	242
Currently in school	19670	2	1	4	6706 (4614)	6707 (4683)	1
Employment status							
Regular worker	674559	64	60	56	6624 (4801)	6833 (4967)	209
Non-regular worker	291626	28	29	29	5706 (4501)	6071 (4822)	365
Executive	34504	3	3	6	6151 (4689)	6254 (4770)	103
Self-employed	57753	5	7	9	5787 (4577)	5948 (4726)	161

Iterative proportional fitting was used to calibrate survey weights to national population margins for age, gender, and household income using the 2020 Population Census and the 2018 Housing and Land Survey. Users with missing household income data were excluded (n = 300,430); the remaining 1,242,106 users from the overall analytical sample were included in this analysis. Educational attainment and employment status were not used for weight calibration; weighted estimates for these variables reflect the indirect effect of age, gender, and income adjustment.

[a] Difference: weighted mean daily steps minus unweighted mean daily steps.

Appendix Table 4. Characteristics of participants in the municipality-level and individual-level analyses

	Municipality-level analysis[a] (n = 1,503,745)			Individual-level analysis[b] (n = 1,297,524)		
	Number of users[c]	Mean steps (SD)	P values	Number of users[c]	Mean steps (SD)	P values
Age, mean (SD)	39·2 (12·1)			40·3 (11·4)		
20–29	410875 (27%)	6305 (7770)	<0·0001[d]	287841 (22%)	6188 (7676)	<0·0001[d]
30–39	370946 (25%)	5953 (7433)		351986 (27%)	5935 (7415)	
40–49	354633 (24%)	6023 (7600)		327632 (25%)	6026 (7604)	
50–59	297246 (20%)	6305 (7965)		268500 (21%)	6337 (7999)	
60–64	70045 (5%)	6614 (8337)		61565 (5%)	6657 (8378)	
Gender						
Men	541301 (36%)	7375 (9020)	<0·0001[e]	445909 (34%)	7497 (9143)	<0·0001[e]
Women	962444 (64%)	5488 (6898)		851615 (66%)	5427 (6838)	
Marital status						
Married	808779 (54%)	5967 (7524)	<0·0001[e]	753862 (59%)	5959 (7517)	<0·0001[e]
Not married	677613 (46%)	6452 (8014)		528601 (41%)	6443 (8026)	
Presence of children						
Having one or more children	677810 (46%)	5780 (7308)	<0·0001[e]	623710 (49%)	5764 (7295)	<0·0001[e]
Not having children	808582 (54%)	6531 (8107)		658753 (51%)	6535 (8125)	
Educational attainment						
University or higher	460782 (31%)	6720 (8214)	<0·0001[d]	410736 (32%)	6729 (8219)	<0·0001[d]
Junior college/Vocational school	416429 (28%)	5738 (7232)		383611 (30%)	5727 (7221)	
High school or less	561644 (38%)	5990 (7650)		488116 (38%)	5959 (7639)	
Currently in school	47537 (3%)	6608 (8009)		NA	NA	
Employment status						
Regular worker	750029 (50%)	6601 (8152)	<0·0001[d]	768382 (59%)	6574 (8128)	<0·0001[d]
Non-regular worker	358898 (24%)	5729 (7283)		367417 (28%)	5701 (7254)	
Non-worker	159145 (11%)	5072 (6582)		161725 (13%)	5052 (6563)	
Executive/Manager	36220 (2%)	6136 (7728)		NA	NA	
Self-employed	64353 (4%)	5783 (7369)		NA	NA	
Student	103009 (7%)	6628 (8018)		NA	NA	
Other (not elsewhere classified)	32091 (2%)	5825 (7415)		NA	NA	
Equivalised household income[f] (Japanese Yen)						
≥5·0 million yen	232926 (19%)	6701 (8221)	<0·0001[d]	199289 (18%)	6740 (8254)	<0·0001[d]
2·5–4·9 million yen	615997 (51%)	6258 (7822)		566002 (52%)	6256 (7821)	
<2·5 million yen	362123 (30%)	5808 (7411)		316609 (29%)	5753 (7366)	

[a] Municipality-level analysis included users aged 20–64 years with valid step count records (≥10 days with 500–29,999 steps/day) who resided in municipalities with at least 100 users per season.

[b] Individual-level analyses included users aged 20–64 years with valid daily step records. Users who reported being "currently in school" (educational attainment) or were classified as "student", "self-employed", "executive/manager", or "other" (employment status) were excluded and coded as NA.

[c] Values are presented as n (%) unless otherwise stated. The user count varied for some variables due to missing data.

Differences in mean daily steps across demographic groups were assessed using [d]ANOVA or [e]t-tests.

[f] Equivalised household income was calculated by dividing annual household income by the square root of household size.

Appendix Table 5. Municipality-level demographic proportions of TRIMA users and the national population

	TRIMA users[a], % (IQR)	National population, % (IQR)	Deviation (TRIMA - National), % (IQR)
Age[b], years			
20–49	76 (73, 79)	63 (60, 66)	12 (9, 16)
Gender[b]			
Women	64 (63, 66)	49 (48, 51)	15 (13, 16)
Educational attainment[c]			
University or higher education	23 (19, 29)	24 (19, 29)	-1 (-2, 1)
Employment status[d]			
Regular employee	62 (59, 64)	56 (53, 58)	6 (4, 9)
Household income[e]			
≥5·0 million yen	53 (48, 58)	34 (29, 39)	19 (16, 21)

For each variable, the proportion was calculated within each municipality for both TRIMA users and the national population; values are the median (IQR) across 951 municipalities, except for household income (939 municipalities for which data were available from the official statistics). For the national population, proportions of age, gender, and education were calculated among those aged 20–64 years; proportions of employment status and household income were not restricted by age due to data availability.

[a] The analysis included users aged 20–64 years with valid step count records (≥10 days with 500–29,999 steps/day), residing in municipalities with ≥100 users. The number of TRIMA users for each variable was as follows: aged 20–49 years, 1,136,454; women, 962,444; university or higher education, 460,782; regular employee, 750,029; and household income ≥5·0 million yen, 655,004.

Japanese national statistics sources:

[b] Portal site of official statistics of Japan (e-Stat), Population census 2020, Basic complete tabulation on population and household, Table number 2–7.

[c] Portal site of official statistics of Japan (e-Stat), Population census 2020, Basic complete tabulation on labour force, Table number 11–2.

[d] Portal site of official statistics of Japan (e-Stat), Population census 2020, Basic complete tabulation on labour force, Table number 5–3.

[e] Portal site of official statistics of Japan (e-Stat), Housing and land survey 2018, Basic tabulation on dwelling and households, Table number 44–4.

Appendix Table 6. Municipality-level summary statistics for the number of users, mean daily steps, and Gini coefficients

Rank	Prefecture	Municipality (ward/city/town)	Number of users	Mean daily steps (SD)	Gini coefficients
1	Tokyo	Toshima Ward	3624	7750 (8591)	0·33
2	Tokyo	Bunkyo Ward	2173	7719 (4762)	0·33
3	Tokyo	Shibuya Ward	2522	7666 (11179)	0·33
4	Chiba	Urayasu City	2918	7647 (9739)	0·35
5	Tokyo	Nakano Ward	4506	7643 (9406)	0·33
6	Tokyo	Shinjuku Ward	4032	7626 (8137)	0·33
7	Tokyo	Koto Ward	6259	7583 (9295)	0·34
8	Tokyo	Taito Ward	2640	7578 (10705)	0·34
9	Tokyo	Shinagawa Ward	5141	7574 (8335)	0·33
10	Tokyo	Minato Ward	2546	7563 (11258)	0·33
942	Ibaraki	Itako City	207	4489 (3701)	0·42
943	Miyazaki	Takanabe Town	213	4470 (3497)	0·41
944	Kagoshima	Minamikyushu City	220	4452 (3577)	0·42
945	Kagoshima	Soo City	206	4441 (3788)	0·43
946	Iwate	Ofunato City	233	4425 (3818)	0·43
947	Fukushima	Kitakata City	278	4405 (3868)	0·44
948	Miyazaki	Nichinan City	402	4305 (3571)	0·42
949	Nagasaki	Minamishimabara City	245	4216 (3416)	0·42
950	Fukuoka	Ukiha City	207	4205 (3560)	0·42
951	Miyazaki	Kobayashi City	314	4026 (3405)	0·43

This table presents the 10 municipalities with the highest and the 10 with the lowest mean daily steps.

Appendix Table 7. Simulation results for hypothetical users' step counts and correlations between simulated municipal mean daily steps and Gini coefficients

Probability distribution	Mean daily steps of hypothetical users (95% CI[a])	SD[b] for hypothetical users' daily steps (95% CI[a])	Correlation coefficient[c] (95% CI[a])
Normal	7162 (7156, 7167)	3877 (3874, 3881)	-0·40 (-0·56, -0·23)
Log-normal	6052 (6046, 6058)	4235 (4228, 4243)	0·32 (0·14, 0·49)
Gamma	6241 (6235, 6247)	4532 (4525, 4539)	0·00 (-0·19, 0·19)

[a] CI: Confidence interval. [b] SD: Standard deviation. [c] Pearson's correlation coefficients were calculated between municipal mean daily steps and Gini coefficients across simulated municipalities.

Appendix Table 8. Summary of users, municipalities, and step-count variability by municipal walkability definition

Definitions of municipal walkability and categories[a]	Users, n (%)	Municipalities, n (%)	IQRs of municipal mean daily steps[b]	Mean (SD) of within-municipality IQRs of users' mean daily steps[c]
Municipal mean values	1503745	951		
C5: 1·56 to 2·24	164869 (11%)	26 (3%)	414	3399 (175)
C4: 0·89 to 1·55	447780 (30%)	91 (10%)	498	3651 (223)
C3: 0·22 to 0·88	515275 (34%)	256 (27%)	784	3651 (270)
C2: -0·45 to 0·21	307208 (20%)	389 (41%)	551	3545 (306)
C1: -1·11 to -0·44	68613 (5%)	189 (20%)	469	3465 (351)
Municipal maximum values	1503745	951		
C5: 2·05 to 2·59	261974 (17%)	30 (3%)	442	3461 (230)
C4: 1·52 to 2·04	448955 (30%)	87 (9%)	512	3676 (214)
C3: 0·98 to 1·51	485395 (32%)	243 (26%)	759	3629 (248)
C2: 0·45 to 0·97	277180 (18%)	468 (49%)	555	3535 (310)
C1: -0·08 to 0·44	30241 (2%)	123 (13%)	481	3488 (403)
Municipal mean of the top five values	1503745	951		
C5: 1·94 to 2·54	274473 (18%)	30 (3%)	471	3457 (227)
C4: 1·34 to 1·93	543791 (36%)	113 (12%)	610	3667 (218)
C3: 0·75 to 1·33	515325 (34%)	366 (38%)	693	3600 (265)
C2: 0·16 to 0·74	158074 (11%)	383 (40%)	562	3520 (329)
C1: -0·43 to 0·15	12082 (1%)	59 (6%)	479	3476 (446)
Municipal mean values (Quintile-based categorisation)	1503745	951		
Q5: 0·64 to 2·24	813944 (54%)	190 (20%)	926	3650 (250)
Q4: 0·21 to 0·63	321174 (21%)	190 (20%)	645	3614 (268)
Q3: -0·16 to 0·20	183077 (12%)	190 (20%)	531	3555 (275)
Q2: -0·45 to -0·15	115507 (8%)	190 (20%)	561	3532 (339)
Q1: -1·11 to -0·44	70043 (5%)	191 (20%)	476	3467 (351)

[a] Municipal walkability was defined using the Japan Postcode-level Walkability Index,[1] aggregated at the municipal level using the listed calculation methods. For each method, municipalities were grouped into five equal-interval categories based on their scores unless otherwise stated.

[b] Interquartile ranges (IQRs) were calculated based on the mean daily steps between municipalities in each walkability category.

[c] Values represent within-municipality variability in step counts, calculated by deriving the IQR of users' mean daily steps within each municipality and then averaging these IQRs within each walkability category. Values in parentheses indicate the standard deviation.

Appendix Table 9. Proportions of missing data for variables included in multiple imputation

Variable	Proportion missing
Gender	0·000
Age	0·000
Marital status	0·012
Household size	0·012
Presence of children	0·012
Educational attainment	0·012
Employment status	0·000
Equivalised household income	0·166
Walkability[a]	0·000

[a] Walkability was defined based on the Japan Postcode-level Walkability Index[1]

Appendix Table 10. Results of linear mixed-effects models for mean daily steps in men

Variables (reference category)	Educational attainment model	Employment status model	Equivalised household income[a] model
**Intercept**	6673 (6616, 6729)***	6690 (6634, 6745)***	6713 (6654, 6771)***
**Age (ref: 20–29)**			
30–39	193 (159, 228)***	191 (157, 226)***	192 (158, 227)***
40–49	210 (175, 245)***	207 (172, 242)***	207 (172, 243)***
50–59	201 (165, 238)***	197 (160, 233)***	196 (160, 233)***
60–64	332 (279, 385)***	328 (275, 380)***	328 (275, 381)***
**Marital status (ref: married)**			
Not married	83 (52, 114)***	81 (50, 112)***	81 (50, 112)***
**Presence of children (ref: None)**			
One or more	117 (87, 146)***	116 (86, 145)***	115 (85, 144)***
**Educational attainment (ref: ≥ University degree)**			
Junior college or Vocational school	-128 (-167, -90)***	-110 (-142, -78)***	-109 (-140, -77)***
≤ High school	68 (37, 99)***	56 (30, 82)***	58 (32, 84)***
**Employment status (ref: Regular worker)**			
Non-regular	450 (410, 490)***	461 (412, 510)***	444 (404, 484)***
Non-worker	-862 (-921, -803)***	-859 (-931, -788)***	-868 (-927, -808)***
**Equivalised household income[a] (ref: ≥5·0 million yen)**			
2·5–4·9 million yen	122 (93, 151)***	113 (84, 143)***	85 (49, 122)***
<2·5 million yen	118 (81, 156)***	112 (74, 150)***	94 (49, 139)***
**Walkability[b] (linear term)**	396 (369, 423)***	325 (302, 349)***	314 (282, 346)***
**Walkability[b] (squared term)**	60 (44, 76)***	71 (59, 83)***	51 (31, 72)***
**Random-effects variance**			
Municipality level (SD[c])	225883 (475)	223411 (473)	223076 (472)
Postcode level (SD[c])	215496 (464)	216344 (465)	216113 (465)
Residual (SD[c])	13547855 (3681)	13548730 (3681)	13550425 (3681)
**P for interaction**	<0·0001	<0·0001	<0·0001
**Intraclass Correlation Coefficients**	0·031 (0·031, 0·031)	0·031 (0·031, 0·031)	0·031 (0·031, 0·031)
**PVE[d] after adding Walkability and interaction terms**			
Municipality level	0.44	0.44	0.45
Postcode level	0.04	0.04	0.04
Residuals	0.00	0.00	0.00

Values are presented as coefficients or estimates (95% confidence intervals) for three separate models, unless otherwise specified. Each column corresponds to a model including interaction terms between a specific socioeconomic status (educational attainment, employment status, or equivalised household income) and walkability (both the linear and squared terms), with adjustments for age, marital status, presence of children, and the other SES indicators not used in the interaction term.

[a] Calculated by dividing annual household income (Japanese yen) by the square root of the household size.

[b] Defined using the Japan Postcode-level Walkability Index[1] and both linear and squared terms were included.

[c] SD: standard deviation of random intercepts, indicating between-group variability.

[d] PVE: proportion of variance explained, calculated as the reduction in the random intercept variance at each level after adding walkability and its interaction terms.

P-values: * p ≤ 0·05, ** p ≤ 0·01, *** p ≤ 0·001.

Appendix Table 11. Results of linear mixed-effects models for mean daily steps in women

Variables (reference category)	Educational attainment Model	Employment status model	Equivalised household income[a] Model
**Intercept**	4701 (4661, 4740)***	4689 (4650, 4727)***	4725 (4684, 4765)***
**Age (ref: 20–29)**			
30–39	-163 (-179, -146)***	-167 (-183, -150)***	-164 (-181, -148)***
40–49	-64 (-82, -46)***	-63 (-81, -45)***	-66 (-84, -49)***
50–59	66 (47, 85)***	68 (50, 87)***	63 (44, 82)***
60–64	143 (112, 174)***	145 (113, 176)***	139 (107, 170)***
**Marital status (ref: married)**			
Not married	417 (403, 432)***	415 (400, 430)***	416 (401, 430)***
**Presence of children (ref: None)**			
One or more	575 (561, 590)***	572 (558, 586)***	575 (561, 589)***
**Educational attainment (ref: ≥ University degree)**			
Junior college or Vocational school	-252 (-271, -233)***	-242 (-257, -227)***	-252 (-267, -237)***
≤ High school	-244 (-263, -224)***	-252 (-268, -237)***	-260 (-276, -245)***
**Employment status (ref: Regular worker)**			
Non-regular	66 (52, 80)***	104 (88, 120)***	64 (50, 78)***
Non-worker	-545 (-562, -527)***	-493 (-514, -472)***	-548 (-565, -530)***
**Equivalised household income[a] (ref: ≥5·0 million yen)**			
2·5–4·9 million yen	-51 (-70, -33)***	-48 (-67, -29)***	-69 (-92, -45)***
<2·5 million yen	-102 (-123, -81)***	-99 (-120, -78)***	-96 (-122, -71)***
**Walkability[b] (linear term)**	404 (386, 421)***	431 (416, 447)***	337 (316, 357)***
**Walkability[b] (squared term)**	69 (58, 79)***	99 (91, 107)***	74 (60, 88)***
**Random-effects variance**			
Municipality level (SD[c])	234943 (485)	228041 (478)	232604 (482)
Postcode level (SD[c])	97274 (312)	97960 (313)	97970 (313)
Residual (SD[c])	6901255 (2627)	6896882 (2626)	6903067 (2627)
**P for interaction**	< 0·0001	< 0·0001	<0·0001
**Intraclass Correlation Coefficients**	0.046 (0.046, 0.046)	0.045 (0.045, 0.045)	0.046 (0.046, 0.046)
**PVE[d] after adding Walkability and interaction terms**			
Municipality level	0.43	0.44	0.43
Postcode level	0.16	0.16	0.16
Residuals	0.00	0.00	0.00

Values are presented as coefficients or estimates (95% confidence intervals) for three separate models, unless otherwise specified. Each column corresponds to a model including interaction terms between a specific socioeconomic status (educational attainment, employment status, or equivalised household income) and walkability (both the linear and squared terms), with adjustments for age, marital status, presence of children, and the other SES indicators not used in the interaction term.

[a] Calculated by dividing annual household income (Japanese yen) by the square root of the household size.

[b] Defined using the Japan Postcode-level Walkability Index[1] and both linear and squared terms were included.

[c] SD: standard deviation of random intercepts, indicating between-group variability.

[d] PVE: proportion of variance explained, calculated as the reduction in the random intercept variance at each level after adding walkability and its interaction terms.

P-values: * p ≤ 0·05, ** p ≤ 0·01, *** p ≤ 0·001.

Appendix Table 12. Model-adjusted mean daily steps by socioeconomic status and walkability categories, stratified by gender

Gender	SES[a] domain	SES[a] category	Walkability categories[b]					C5-C1 Diff[c]
			C5	C4	C3	C2	C1	
Men	Educational attainment	University degree or higher	7464	7187	6983	6782	6436	**1028**
		Junior college/Vocational school	7295	7057	6883	6711	6415	880
		High school or less	7355	7169	7032	6898	6666	689
	Employment status	Regular worker	7513	7285	7117	6953	6668	845
		Non-regular worker	8117	7797	7561	7330	6930	**1187**
		Non-worker	6603	6399	6248	6100	5844	759
	Equivalised household income[d]	≥5·0 million yen	7286	7066	6904	6745	6471	815
		2·5–4·9 million yen	7444	7198	7018	6840	6533	**911**
		<2·5 million yen	7416	7184	7013	6845	6555	861
Women	Educational attainment	University degree or higher	5562	5280	5072	4868	4515	**1047**
		Junior college or Vocational school	5281	5024	4834	4649	4328	953
		High school or less	5138	4948	4807	4669	4431	707
	Employment status	Regular worker	5610	5308	5086	4867	4490	**1120**
		Non-regular worker	5499	5292	5140	4990	4732	767
		Non-worker	4784	4635	4526	4418	4233	551
	Equivalised household income[d]	≥5·0 million yen	5382	5147	4974	4803	4509	873
		2·5–4·9 million yen	5343	5097	4917	4739	4433	**910**
		<2·5 million yen	5271	5037	4865	4695	4403	868

Values represent adjusted mean daily steps within each socioeconomic status (SES) group and walkability category. Estimates were derived from linear mixed-effects models including interaction terms between SES and walkability (both the linear and squared terms), adjusted for age, marital status, presence of children, and the other SES indicators not used in the interaction term.

[a] SES: Socioeconomic status.

[b] Walkability was defined using the Japan Postcode-level Walkability Index[1] and categorised into quintiles as follows: C1 (lowest: -1·85 to 0·36), C2 (0·37 to 0·76), C3 (0·77 to 1·09), C4 (1·10 to 1·47), and C5 (highest: 1·48 to 2·59). The median walkability value within each quintile was used to estimate adjusted mean daily steps.

[c] C5-C1 Diff: differences in adjusted mean daily steps between C5 and C1 within each SES subgroup (bold = the maximum difference across SES subgroups within each SES domain).

[d] Calculated by dividing annual household income (Japanese yen) by the square root of the household size.

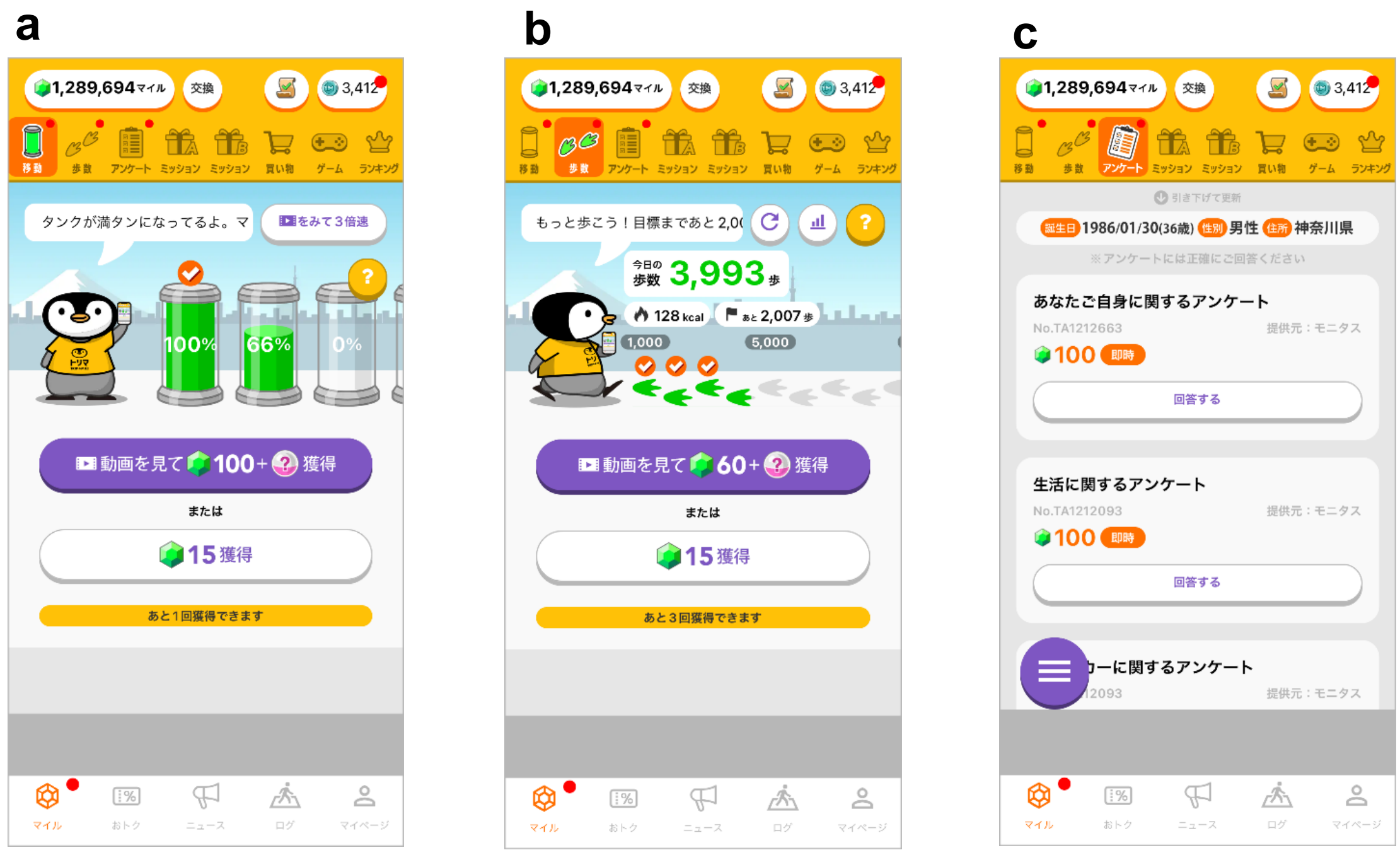

Appendix Figure 1. Screenshots of the TRIMA app

TRIMA is a point-based app that allows users to earn points through various activities, including distance travelled, step counts, and questionnaire completion. The images illustrate point-earning screens for: (a) distance travelled, (b) step counts, and (c) questionnaires in Japanese.

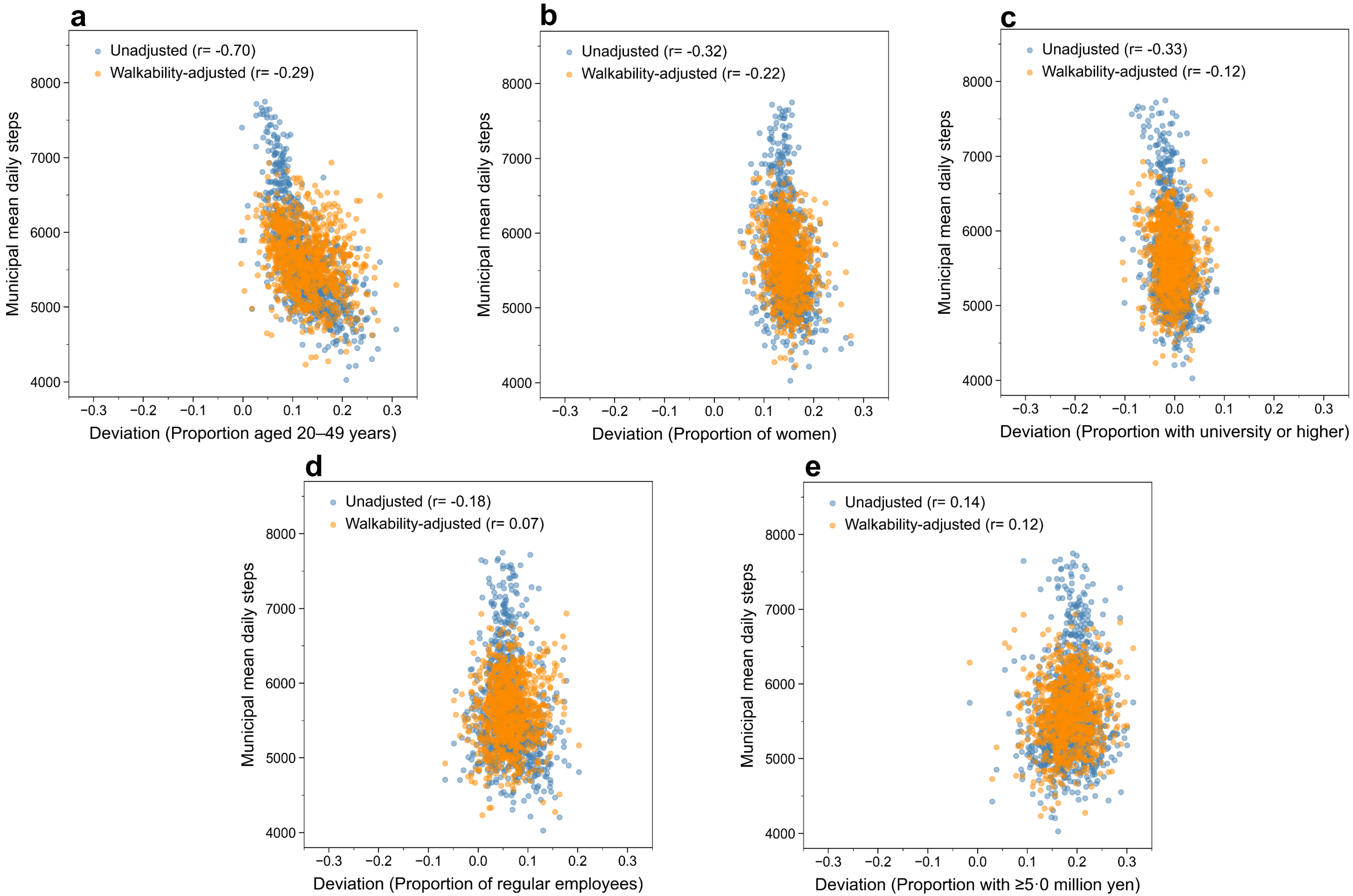

Appendix Figure 2. Association between municipality-level demographic deviations and mean daily steps, before and after adjustment for walkability

Each panel shows the deviation in the proportion of a demographic subgroup (TRIMA users minus national population) and municipality-level mean daily steps for 951 municipalities (939 for household income). Blue dots represent unadjusted mean daily steps; orange dots represent walkability-adjusted mean daily steps using ordinary least squares regression. Walkability was defined as the municipal mean of the Japan Postcode-level Walkability Index.[1] Pearson's correlation coefficients (r) are shown in each panel. (a) Age 20–49 years (n = 951 municipalities), (b) women (n = 951), (c) university or higher education (n = 951), (d) regular employee (n = 951), and (e) household income ≥ 5·0 million yen (n = 939).

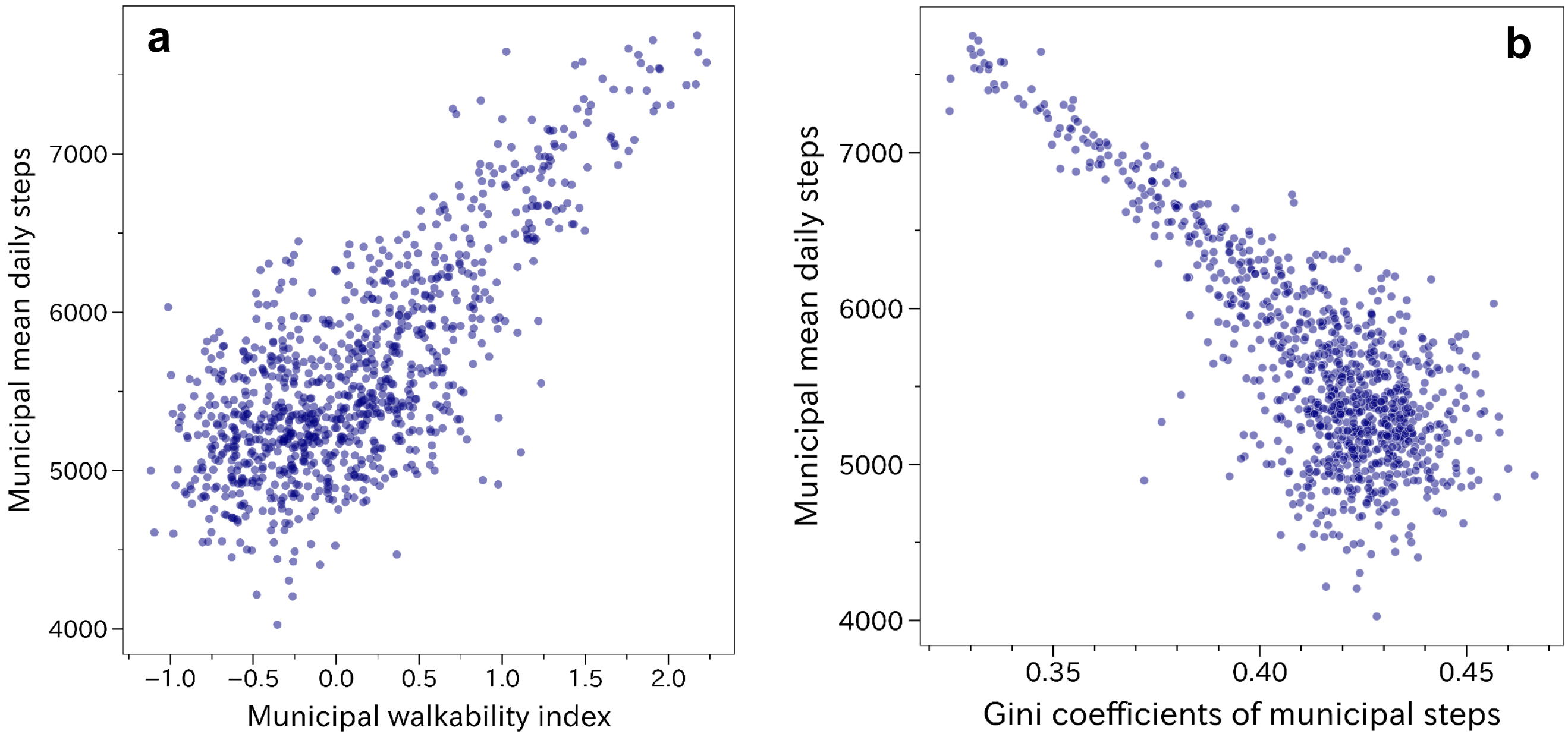

Appendix Figure 3. Associations of municipal mean daily steps with municipal walkability and Gini coefficients

Each dot represents one of the 951 out of 1,747 municipalities in Japan included in the municipality-level analysis. The scatter plots show the associations between municipal mean daily steps and (a) the municipal walkability (r = 0·76) and (b) the Gini coefficient of step counts (r = -0·79). The municipal walkability was defined based on the municipal mean of the Japan Postcode-level Walkability Index.[4]

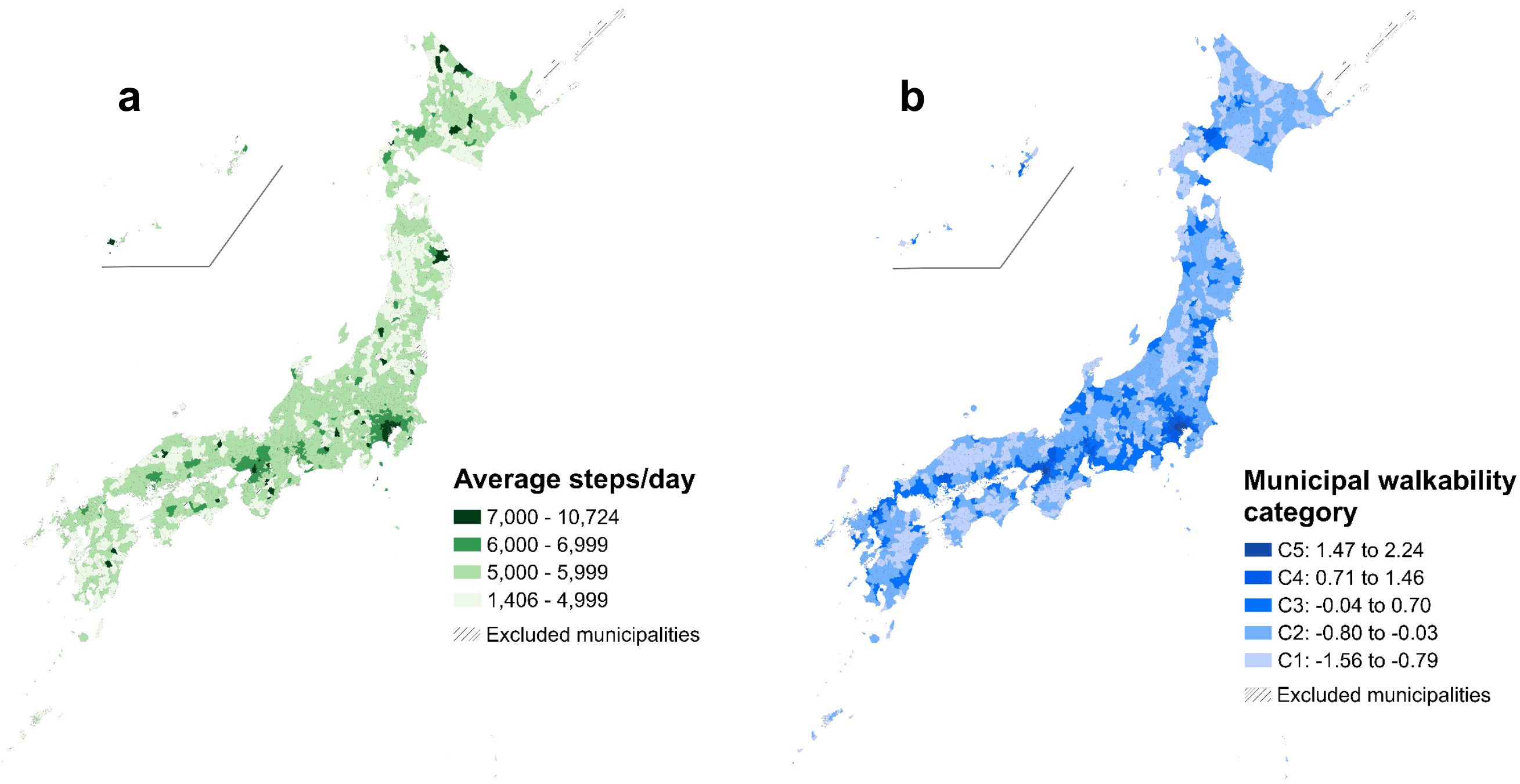

Appendix Figure 4. Nationwide geographical distribution of municipal mean daily steps and walkability

These maps illustrate the nationwide geographical distribution of municipality-level variables.

(a) The distribution of municipal mean daily steps across 1,727 municipalities (n = 1,542,536 users). Users were aged 20–64 years and had ≥10 days of valid step data (500–29,999 steps/day). No minimum user threshold was applied at the municipality level.

(b) The distribution of municipal walkability across 1,741 municipalities, categorised into five equal-width intervals. Municipal walkability was defined as the municipal mean of the Japan Postcode-level Walkability Index.[1]

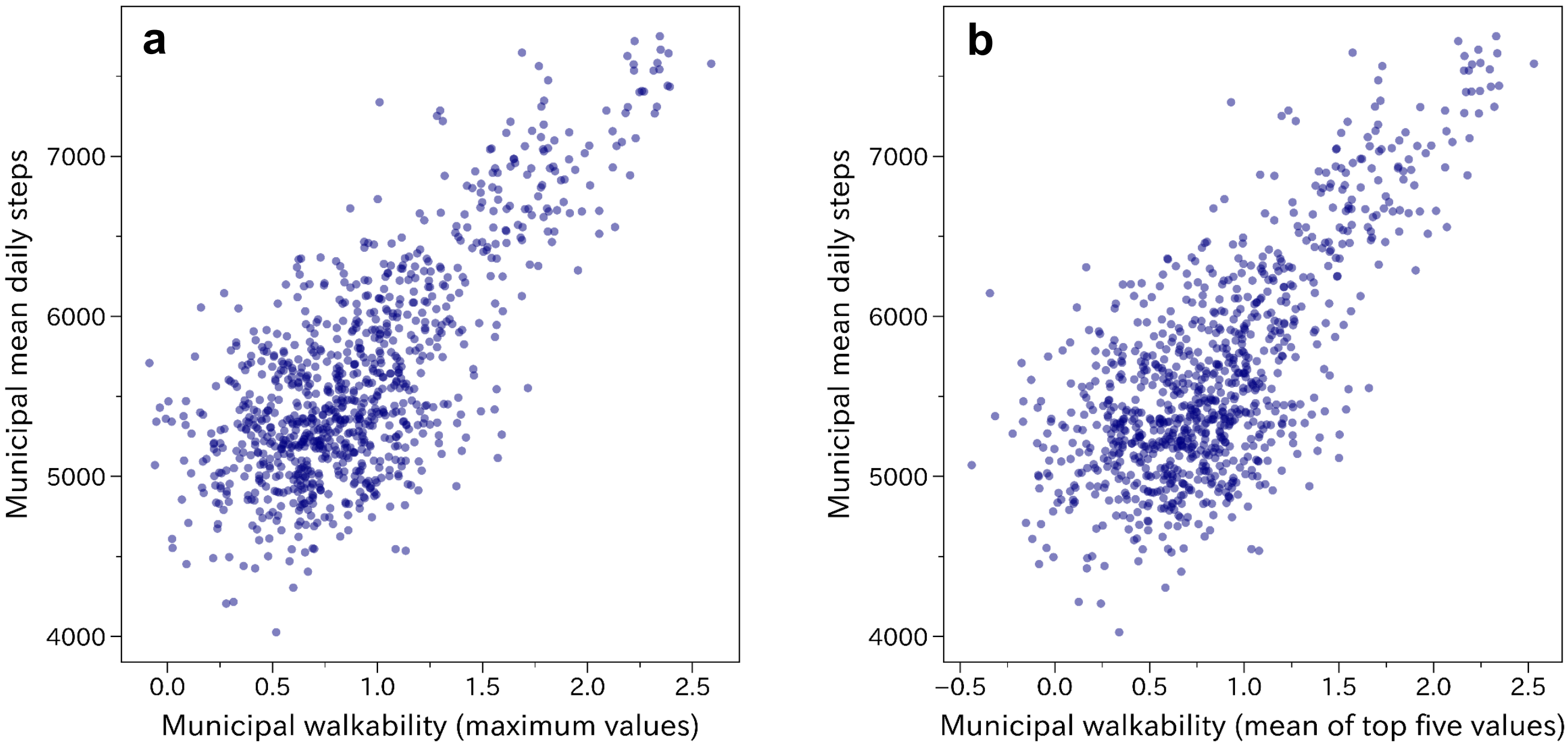

Appendix Figure 5. Association between municipal mean daily steps and alternative definitions of municipal walkability

Each dot represents one of the 951 out of 1,747 municipalities in Japan included in the municipality-level analysis. The scatter plots illustrate the associations between municipal mean daily steps and two alternative definitions of municipal walkability derived from the Japan Postcode-level Walkability Index[1]: (a) the maximum value and (b) the mean of the top five values within each municipality.

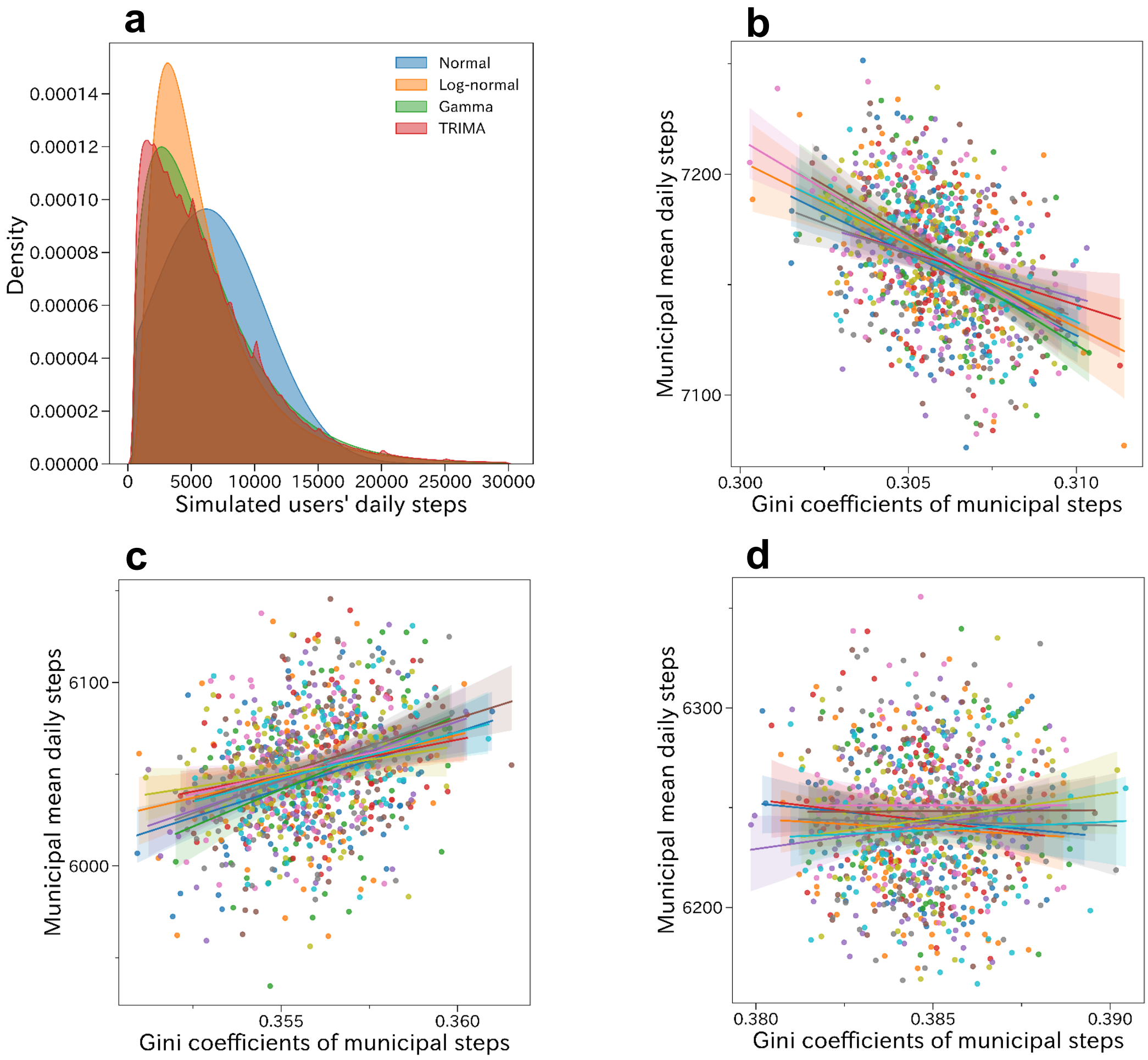

Appendix Figure 6. Simulated distributions of user- and municipality-level step counts from 5,000 simulations

(a) Distributions of simulated users' daily steps generated from normal, log-normal, and gamma distributions, compared with the observed user data. For visualisation purposes, distributions are shown for random 500 iterations because of computational constraints. (b–d) Associations between simulated municipal mean daily steps and Gini coefficients of population step counts for the (b) normal, (c) log-normal, and (d) gamma distributions. Pearson correlation coefficients with 95% confidence intervals (r [95% CI]) are shown for each distribution: normal (-0·40 [-0·56, -0·23]), log-normal (0·32 [0·14, 0·49]), and gamma (0·00 [-0·19, 0·19]). Each coloured dot and line represents a different iteration (10 randomly selected iterations are displayed), and each dot represents one municipality. Solid lines indicate linear regression fits, and shaded areas denote 95% confidence intervals.

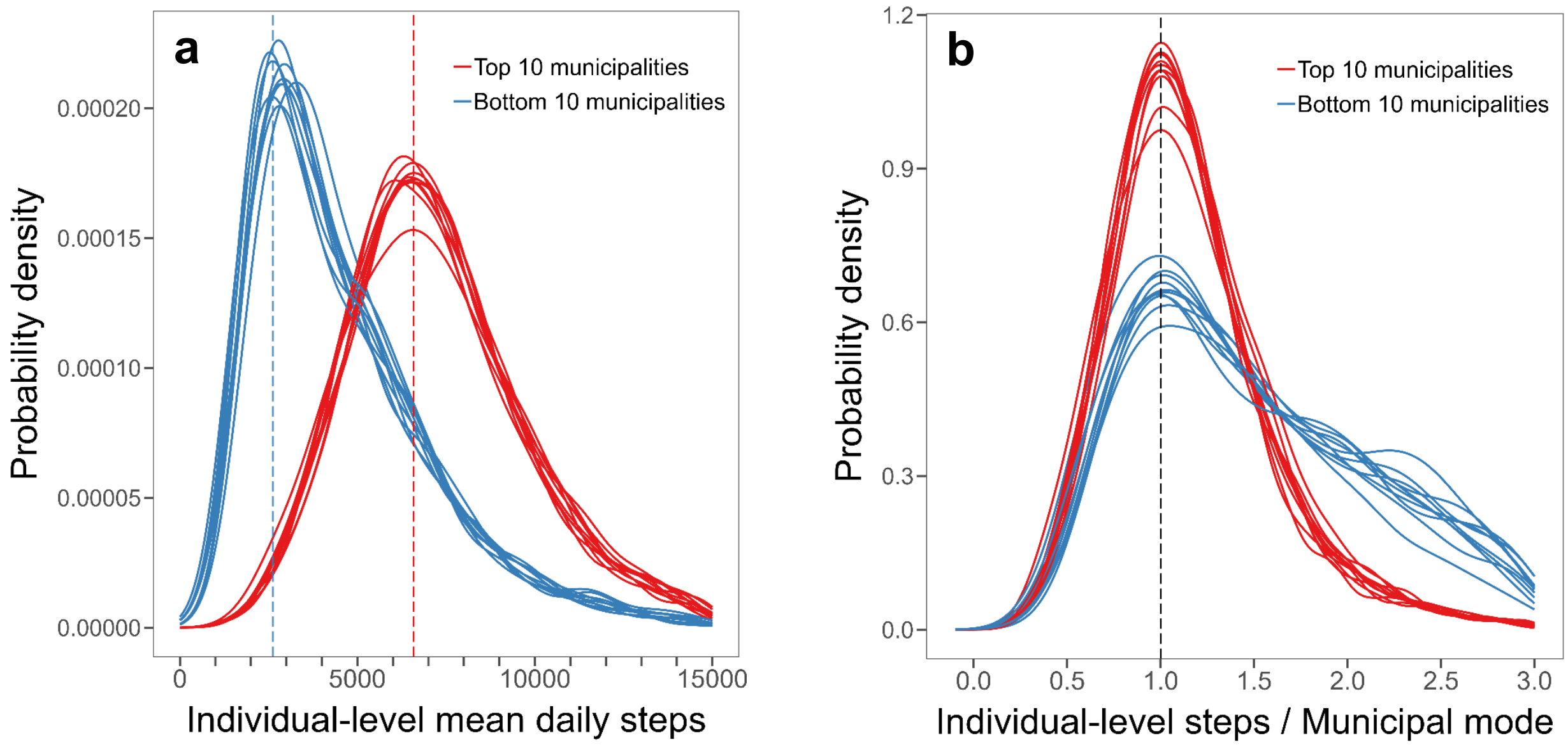

Appendix Figure 7. Within-municipality distributions of individual-level mean daily steps (municipalities with ≥1,000 users)

Panels (a) and (b) show distributions of individual-level mean daily steps in the top 10 (red) and bottom 10 (blue) municipalities ranked by municipal mean daily steps, among municipalities with at least 1,000 users. Panel (a) shows raw distributions, with red and blue dashed lines indicating the mode for Toshima Ward, Tokyo (the highest-step municipality) and Ishinomaki City, Miyagi (the lowest-step municipality), respectively. Panel (b) shows mode-normalised distributions, with the municipal mode indicated by the black dashed line.

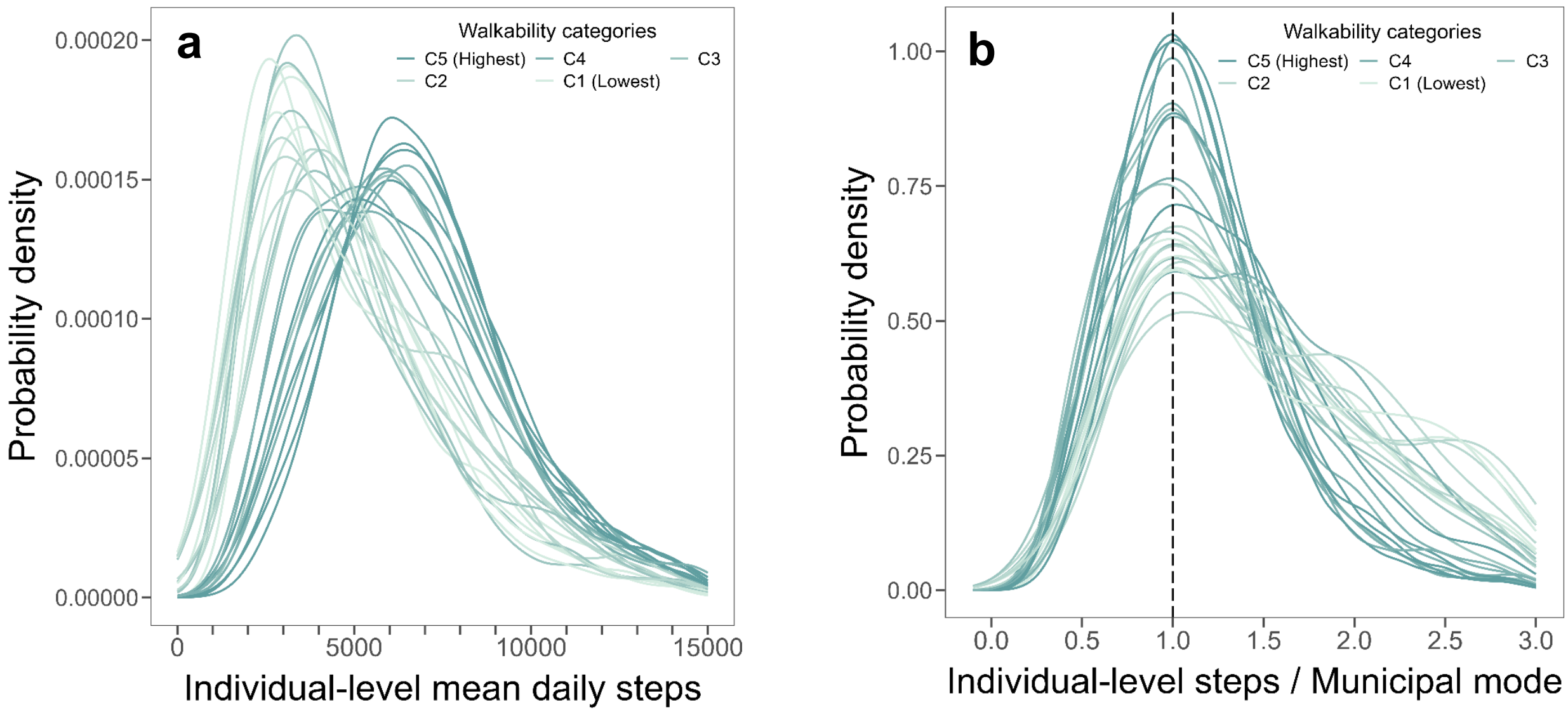

Appendix Figure 8. Within-municipality distributions of individual-level mean daily steps based on municipal walkability categories

Panels (a) and (b) show distributions of individual-level mean daily steps across 25 municipalities (five randomly selected from each municipal walkability category). Municipal walkability was defined as the mean of the Japan Postcode-level Walkability Index[1] and stratified into five equal-width intervals: C1 (-1·11 to -0·44), C2 (-0·45 to 0·21), C3 (0·22 to 0·88), C4 (0·89 to 1·55), and C5 (1·56 to 2·24). Panel (a) shows raw distributions and panel (b) shows mode-normalised distributions, with the municipal mode indicated by the black dashed line.

References

1. Tanimoto R, Hanibuchi T, Nakaya T. Development of Japan postcode-level walkability index and examination of its usefulness. *Q J Geogr* 2023; **75**: 16–26.
2. Atkinson AB. On the measurement of inequality. *J Econ Theory* 1970; **2**: 244–63.
3. Ministry of Health, Labour and Welfare. The National Health and Nutrition Survey in Japan, 2012. p. 50, 165. https://www.mhlw.go.jp/bunya/kenkou/eiyou/dl/h24-houkoku.pdf (accessed December 4, 2025) [in Japanese].
4. Ministry of Health, Labour and Welfare. The National Health and Nutrition Survey in Japan, 2016. p. 37, 161. https://www.mhlw.go.jp/content/001066497.pdf (accessed December 4, 2025) [in Japanese].
